# Assessment of the Retina of Plp-α-Syn Mice as a Model for Studying Synuclein-Dependent Diseases

**DOI:** 10.1167/iovs.61.6.12

**Published:** 2020-06-05

**Authors:** Kathrin Kaehler, Hartwig Seitter, Adolf M. Sandbichler, Bettina Tschugg, Gerald J. Obermair, Nadia Stefanova, Alexandra Koschak

**Affiliations:** 1 Institute of Pharmacy, Pharmacology and Toxicology, University of Innsbruck, Innsbruck, Austria; 2 Institute of Zoology, University of Innsbruck, Innsbruck, Austria; 3 Department of Physiology and Medical Physics, Medical University Innsbruck, Innsbruck, Austria; 4 Physiology Division, Karl Landsteiner University of Health Sciences, Krems, Austria; 5 Division of Neurobiology, Department of Neurology, Medical University Innsbruck, Innsbruck, Austria

**Keywords:** retina, multiple system atrophy, immunohistochemistry

## Abstract

**Purpose:**

Synucleinopathies such as multiple system atrophy (MSA) and Parkinson's disease are associated with a variety of visual symptoms. Functional and morphological retinal aberrations are therefore supposed to be valuable biomarkers for these neurodegenerative diseases. This study examined the retinal morphology and functionality resulting from human α-synuclein (α-Syn) overexpression in the transgenic Plp-α-Syn mouse model.

**Methods:**

Immunohistochemistry on retinal sections and whole-mounts was performed on 8- to 11-week-old and 12-month-old Plp-α-Syn mice and C57BL/6N controls. Quantitative RT-PCR experiments were performed to study the expression of endogenous and human α-Syn and tyrosine hydroxylase (TH). We confirmed the presence of human α-Syn in the retina in western blot analyses. Multi-electrode array (MEA) analyses from light-stimulated whole-mounted retinas were used to investigate their functionality.

**Results:**

Biochemical and immunohistochemical analyses showed human α-Syn in the retina of Plp-α-Syn mice. We found distinct staining in different retinal cell layers, most abundantly in rod bipolar cells of the peripheral retina. In the periphery, we also observed a trend toward a decline in the number of retinal ganglion cells. The number of TH+ neurons was unaffected in this human α-Syn overexpression model. MEA recordings showed that Plp-α-Syn retinas were functional but exhibited mild alterations in dim light conditions.

**Conclusions:**

Together, these findings implicate an impairment of retinal neurons in the Plp-α-Syn mouse. The phenotype partly relates to retinal deficits reported in MSA patients. We further propose the suitability of the Plp-α-Syn retina as a biological model to study synuclein-mediated mechanisms.

Within the group of neurodegenerative diseases, multiple system atrophy (MSA) is among the most severe and rapidly progressing of them, thus constituting a massive burden for patients and their social environment. With a prevalence of 3.4 to 4.9 cases per 100,000 of a population and an average age of onset of 53 years MSA leads to severe movement and autonomic dysfunction.^1^^–^^3^ MSA belongs to a group of α-synucleinopathies characterized by misfolding and spreading of α-synuclein (α-Syn). This highly conserved presynaptic protein, which is encoded by the *SCNA* gene,^4^ is thought to play a role in synaptic vesicle supply and maintenance of neurotransmitter release at presynaptic terminals^5^^,^^6^ but it is also found in neuronal cell bodies.^7^^,^^8^ A pathophysiological hallmark of the disease is the presence of α-Syn aggregates in oligodendroglia cells, referred to as glial cytoplasmic inclusions (GCIs),^9^^–^^14^ and neurons,^15^ finally leading to neuroinflammation, neurodegeneration, and cell death.^6^^,^^16^

Several genetic models show typical cellular signs of MSA.^13^^,^^14^^,^^17^^–^^22^ Fellner and colleagues^24^ concluded that the myelin proteolipid protein (Plp)-α-Syn transgenic model^23^ reflects all main features of the human MSA pathology. In addition to the motor phenotype, human MSA characteristics such as cardiovascular autonomic dysfunction,^25^^,^^26^ bladder dysfunction,^27^ and sleep impairment^28^ underline the high translatability of this model. Moreover, studies testing new therapeutic approaches have already been performed with Plp-α-Syn mice (reviewed in Stefanova and Wenning^29^).

Visual symptoms of major central nervous system disorders that affect the brain and the spinal cord often precede the conventional diagnosis of these disorders.^30^ Morphological and functional changes in the retina have been described for various α-synucleinopathies. In MSA, significant thinning of retinal layers has been shown,^31^ and recently a correlation between perifoveal retinal thinning and clinical severity has been reported in MSA patients.^32^ Retinal thinning progresses with duration of the disease and is accompanied by a selective loss of ganglion cells in the peripheral retina of MSA patients.^44^^,^^51^ With regard to Parkinson's disease (PD), retinal layer thinning,^33^ α-Syn aggregates, and Lewy bodies have been found in the inner retina of PD patients,^31^^,^^34^^,^^35^ distinct from what has been shown in aged human retinas.^36^ Moreover, loss of contrast sensitivity has been reported in PD^37^^–^^39^ but not in MSA patients.^40^^,^^41^

In order to investigate the retina as a candidate biomarker we examined Plp-α-Syn mice and found that this mouse model partly recapitulated the retinal characteristics of MSA patients. Human α-Syn accumulated in retinal second-order neurons but only slightly affected retinal function. Together, these findings make the retina of Plp-α-Syn mice an interesting model for exploring the biology of α-synucleinopathies.

## Material and Methods

### Animals

Wild-type C57BL/6N and Plp-α-Syn mice^23^ were housed in groups of two to six per cage under standard laboratory conditions (12-hour light/dark cycle) with access to water and food ad libitum. Genotyping was performed as previously described.^42^ Mice of both sexes were investigated at the ages of 8 to 10 weeks (adult) and 1 year (aged). All experiments were performed according to the ethical guidelines with the permission of the Austrian Federal Ministry of Science and Research (breeding permission BMWF-66.011/0120-II/3b/2013).

### Immunohistochemistry

Fixation, embedding, and immunolabeling of vertical sections were performed as previously described.^43^ Retinas were embedded in optical cutting temperature medium (Tissue-Tek O.C.T Compound; Sakura Finetek, Tokyo, Japan). For whole-mount staining, primary antibodies (Table 1) were diluted in an antibody solution comprised of 1% BSA in 1% Triton X-100 (Sigma-Aldrich, St. Louis, MO, USA) and 0.02% sodium azide in 1× PBS, and incubated for 1 week on a shaker at room temperature. Whole-mounts were washed three times in 1× PBS for 30 minutes. Secondary antibodies (Table 2) were diluted in 1× PBS with Tween 20 (Sigma-Aldrich) and incubated overnight at room temperature on a shaker. After additional washing steps, whole-mounts were flattened by cutting them four times (“clover-leaf” cuts) and were mounted using Aqua Poly/Mount (Polysciences, Inc., Warrington, PA, USA).

**Table 1.** Primary Antibodies

Primary Antibodies	Dilution	Company	Order Number
Anti-human-α-synuclein (rat)	1:150	Enzo Life Sciences (Farmingdale, NY, US)	ALX-804-258-L001
Anti-mouse-α-synuclein (rabbit)	1:500	Cell Signaling Technology (Danvers, MA, USA)	4179
Anti-tyrosine hydroxylase (rabbit)	1:5000	MilliporeSigma (Burlington, MA, USA)	AB152
Anti-calbindin D-28K (mouse)	1:10000	Swant (Marly, Switzerland)	300
Anti-PKCα (rabbit)	1:400	Santa Cruz Biotechnology (Dallas, TX, USA)	Sc208
Anti-PLP (rabbit)	1:400	Abcam (Cambridge, UK)	Ab23486
Anti-GFAP (rabbit)	1:1000	Abcam (Cambridge, UK)	ab7260
Anti-RBPMS (guinea pig)	1:500	PhosphoSolutions (Aurora, CO, USA)	1832-RBPMS
Anti-phospho-α-Syn (rabbit)	1:1000	Abcam (Cambridge, UK)	AB51253
Anti-Iba1 (rabbit)	1:750	GeneTex (Irvine, CA, USA)	GTX100042
Anti-pan synuclein (pan α-Syn, mouse)	1:1000	BD Biosciences (San Jose, CA, USA)	610787

**Table 2.** Thermo Fisher Scientific Secondary Antibodies

Secondary Antibodies	Dilution	Order Number
Alexa Fluor 488 Donkey anti-Rabbit IgG (H+L)	1:400	A21206
Alexa Fluor 568 Goat anti-Mouse IgG (H+L)	1:400	A11004
Alexa Fluor 488 Goat anti-Mouse IgG (H+L)	1:400	A11001
Alexa Fluor 568 Goat anti-Rabbit IgG (H+L)	1:400	A11008
Alexa Fluor 568 Goat anti-Guinea Pig IgG (H+L)	1:400	A11075
Alexa Fluor 488 streptavidin conjugate	1:400	S11223
Alexa Fluor 594 Donkey anti-Rat IgG (H+L)	1:400	A21209

For confocal microscopy and image analysis, sections were imaged with a confocal laser scanning microscope (Leica TCS SP5-II; Leica Microsystems, Wetzlar, Germany) at 40× magnification (numerical aperture [NA] = 1.30). A series of micrographs were taken at 0.42-µm intervals and collapsed to a *z*-projection with maximum intensities in ImageJ (National Institutes of Health, Bethesda, MD, USA). The analysis of the retinal layer thickness and cell counting was conducted using ImageJ.

Colocalization analysis of Plp and human α-Syn in the optic nerve was performed for individual slices in each image stack using the Coloc 2 plugin for ImageJ. To increase specificity of the colocalization results, two distinct regions of interest were compared and image stacks were deconvolved beforehand in order to reduce background and noise using the Huygens software suite (Scientific Volume Imaging BV, Hilversum, The Netherlands).

For glial fibrillary acidic protein (GFAP) quantification, we obtained maximum intensity projections of the stacks; the regions of interest (ROIs) contained the area between the outer plexiform layer (OPL) and the ganglion cell layer (GCL) in the central and the peripheral retina. A GFAP-positive signal was segmented using the interactive pixel classification routine in Ilastik software.^44^ Segmented areas were quantified with ImageJ and calculated as GFAP-positive area (% of ROI).

Iba1^+^ microglial cells were analyzed for processes and cell body area. We obtained maximum intensity projections of image stacks from the central, dorsal, and ventral GCL and inner plexiform layer (IPL), as well as the OPL, and used them to train a pixel classification algorithm in the Ilastik software. The algorithm distinguished between processes and cell bodies over a wide range of input images. Measurements from the segmented images were extracted with macros in Image J.

### Retinal Microelectrode Array Recordings

Retina preparation and recordings were performed as described previously.^45^ Dorsal retinas were recorded on a perforated 120-electrode microelectrode array (120pMEA100/30iR-Ti-pr; Multichannel Systems, Reutlingen, Germany) at 30°C in a carbogen-bubbled bath solution (110-mM NaCl, 2.5-mM KCl, 1-mM CaCl_2_, 1.6-mM MgCl_2_, 10-mM d-glucose, and 22-mM NaHCO_3_).

#### Light Stimulation

 The retina was stimulated with grayscale (luminance but not spectral content modulated) visual stimuli by a computer-controlled digital light processing projector (Light Crafter E4500MKII; EKB Technologies, Ltd., Bat Yam, Israel), using the built-in blue and green LED to reflect the rhodopsin and M opsin spectra. The projector image was focused onto the photoreceptors through a tube lens (AC254-050-A; Thorlabs, Newton, NJ, USA) and a 10× water-immersion objective (UMPLFLN10XW, NA = 0.3; Olympus, Tokyo, Japan). We limited the stimulus projector output to a ±50% Weber contrast range around a mean background (gray value, 200 ± 48). The light path contained a Thorlabs 650-nm long-pass dichroic mirror (DMLP650R), a Thorlabs shutter (SH1/M), and two motorized Thorlabs filter wheels (FW102C), with two sets of neutral density (ND) filters (63-390, 63-393, 63-395; Edmund Optics, York, UK) with optical densities from 1 (ND1, 10^1^-fold attenuation) to 3 (ND3, 10^3^-fold attenuation), and a fixed ND3 filter that could be combined.

#### Ex Vivo ERG Signals

Data were low-pass filtered (300 Hz, fourth-order Butterworth filter) and downsampled to 1 kHz. Noisy electrodes were discarded, and the remaining electrodes were averaged for the analysis of ex vivo ERG responses.^46^^,^^47^

#### Ganglion Cell Spiking Activity

Spike sorting and analysis were performed as described previously,^48^ based on the spiking responses of individual units. The response polarity of cells was assigned based on spike counts during the first 450 ms after flash onsets and offsets.^49^ Peaks during preferred polarity flashes were detected using the MATLAB function peakfinder (MathWorks, Natick, MA, USA).

#### Full-Field Stimulation Protocols

Full-field flashes consisted of 1-second negative and positive contrast steps of various contrast magnitudes (2% to 50% Weber contrast) with 5 seconds of background gray (gray value 200) in between. We analyzed the maximum positive responses after either the bright or the dark flash. The negative and positive deflections correspond roughly to in vivo ERG a- and b-waves, respectively. Chirp stimuli consisted of sinusoids with 8 seconds of contrast modulation (0%–50% contrast) at 2 Hz, a 2-second period of background gray (gray value 200) followed by 8 seconds of increasing frequency modulation (0–8 Hz) at 50% contrast. All responses were normalized to the negative deflection upon the 50% bright flash (corresponding to an in vivo ERG a-wave).

### Quantitative Real-Time PCR

#### RNA Extraction and cDNA Synthesis

RNA isolation and cDNA synthesis from mouse retina of wild-type (n = 4) and Plp-α-Syn (n = 5) mice were performed using the RNeasy Lipid Tissue Mini Kit (50) (QIAGEN, Hilden, Germany) and Maxima H Minus First Strand cDNA Synthesis Kit (Thermo Fisher Scientific, Waltham, MA, USA).

#### Quantitative Real-Time PCR

All experiments were carried out using an Applied Biosystems 7500 Fast Real-Time PCR System (Applied Biosystems, Foster, CA, USA) and analyzed as described previously.^50^ The following prefabricated Thermo Fisher Scientific Taqman assays were used: Mm00447333_m1 (mouse α-Syn), Hs 00240907_m1 (human α-Syn), Mm00447557_m1 (Th), and Sdha (Mm01352366_m1).

### Western Blot Analysis

Membrane protein preparation from adult mouse retinas (pools of ten retinas) and brain was performed as described previously.^51^ SDS-PAGE and the western blot procedure were used as previously reported,^52^ except that we used primary and secondary antibodies as indicated in Tables 1 and 2.

### Statistical Analysis

Data are presented as mean ± SEM for the indicated number of experiments (n) from the indicated number of animals (N). If not stated otherwise, statistical analyses of immunohistochemical and qRT-PCR data were performed using the Student's *t*-test. Multi-electrode array (MEA) data were analyzed for the indicated number of retinas (N retinas for ex vivo ERG) or spiking units (n units for ganglion cell activity). Each data point, therefore, represents the averaged signal of a recording from one retina (N, ERG) or the spiking activity of one unit (n, ganglion cell) extracted from one of the ERG recordings. Statistical analysis was performed using the Wilcoxon rank-sum test (MATLAB) for pair-wise comparison because data were not always normally distributed.

## Results

In MSA patients, a reduced number of ganglion cells and thinning of retinal layers have been reported.^53^ We therefore set out to investigate changes in the retina of mice that overexpress human α-Syn under the control of Plp-α-Syn to model MSA.

**Figure 1.**
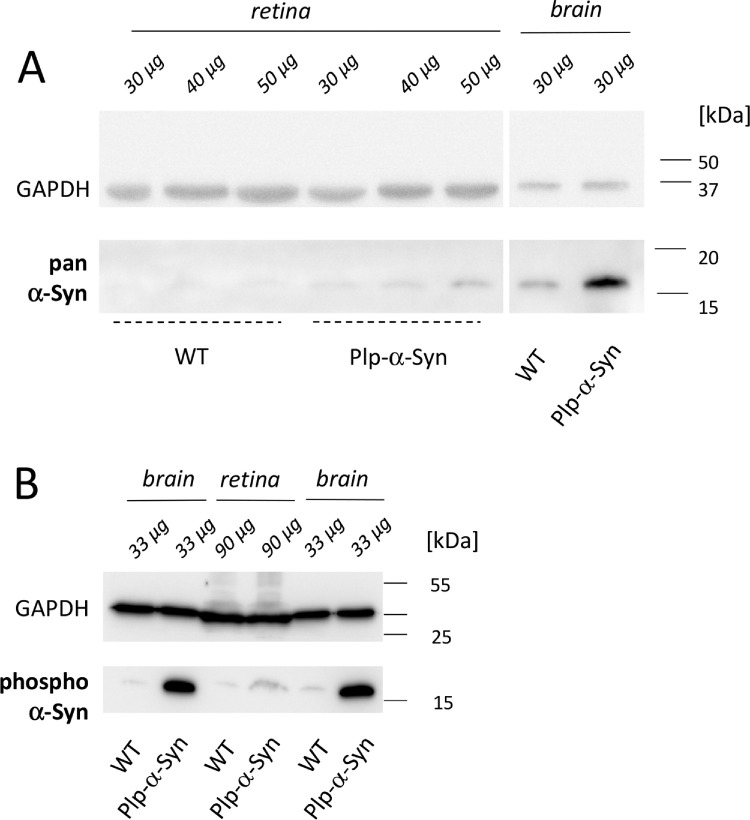
α-Syn in mouse retinas. α-Syn expression was higher in Plp-α-Syn as compared to wild-type (WT) samples. **(A)** Retina of both WT and Plp-α-Syn mice compared to brain positive controls. The antibody (pan α-Syn; see Table 1) detected the murine and human forms of α-Syn. **(B)** The presence of the phosphorylated α-Syn (S129) in the retina, with glyceraldehyde 3-phosphate dehydrogenase (GAPDH) as loading control. In all experiments, the indicated amounts of retinal and brain tissue were loaded. The age of animals was 8 to 10 weeks. The figure shows representative examples of three experiments.

**Figure 2.**
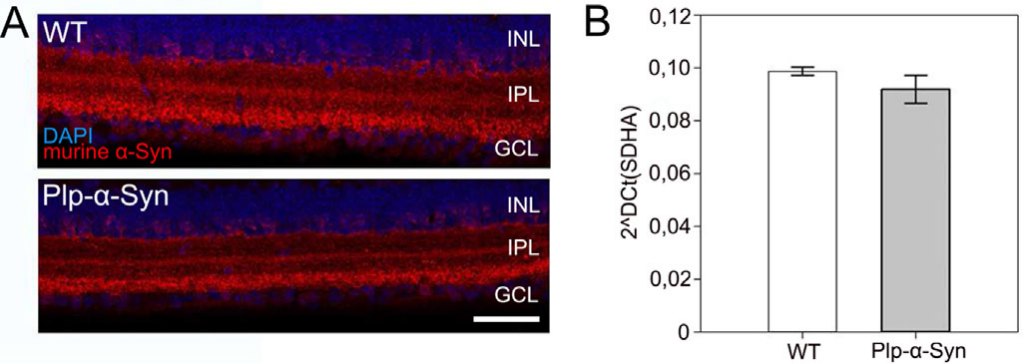
Endogenous α-Syn in wild-type (WT) and Plp-α-Syn retinas. (**A)** Immunoreactivity against murine α-Syn (*red*) in adult WT and Plp-α-Syn mice in the central retina. The antibody exclusively detected endogenous α-Syn (for antibody information, see in Table 1). *Scale bar*: 50 µm. **(B**) Expression levels of murine α-Syn mRNA. Data are presented as mean ± SEM (N = 3 for WT and N = 4 for Plp-α-Syn retinas). The age of animals was 8 to 10 weeks. Statistical analysis was performed using the Student's *t*-test (*P* = 0.338).

We identified human α-Syn in retinas of Plp-α-Syn mice at the ages of 8 to 10 weeks in western blot experiments using a pan α-Syn antibody that detects both human and murine variants of α-Syn (Fig. 1A). Immunoreactivity in the Plp-α-Syn samples indicated an increase of total α-Syn (Fig. 1A). Similar to brain, phosphorylated α-Syn was observed in wild-type and Plp-α-Syn retinas when we used an antibody that detected only α-Syn phosphorylated on Ser129 (Fig. 1B). Expression of the endogenous form of α-Syn was comparable in wild-type and Plp-α-Syn mice, as demonstrated by immunohistochemical analyses (Fig. 2A). qRT-PCR experiments performed with exon-spanning Taqman assays showed that mouse α-Syn mRNA levels also did not differ (Fig. 2B). Human α-Syn mRNA was only detectable in Plp-α-Syn retinas (Supplementary Fig. S1). We assumed that the increase of total α-Syn seen in our western blot analyses was due to the presence of the human form of α-Syn in this mouse model rather than an effect on endogenous α-Syn.

**Figure 3.**
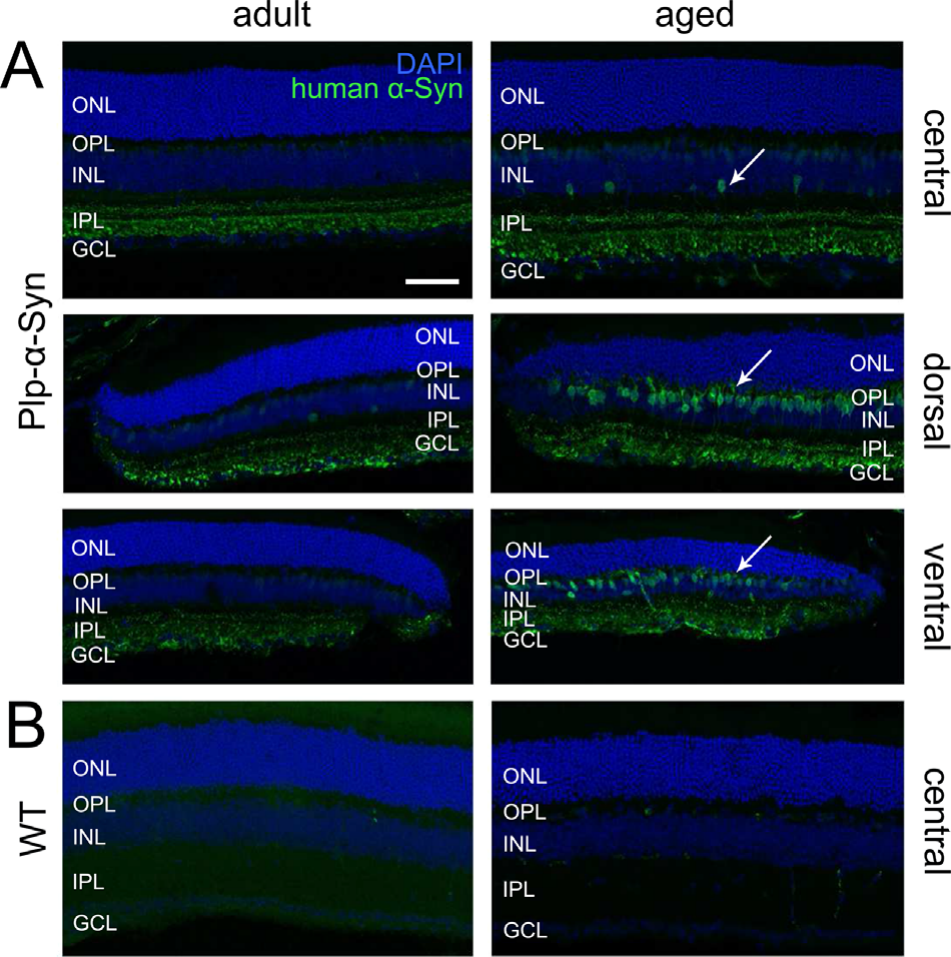
α-Syn immunoreactivity in retinas of different ages. Human α-Syn immunoreactivity (*green*) and 4′,6-diamidino-2-phenylindole (DAPI) (*blue*) of wild-type (WT) and Plp-α-Syn retinal sections. Distinct α-Syn immunoreactivity was obvious in the IPL and GCL in both age groups of Plp-α-Syn but was absent in WT mice. (**A**) Central and peripheral retina of adult (8 weeks) and aged (12 months) Plp-α-Syn animals. The antibody exclusively detected human α-Syn (anti-human-α-synuclein; see Table 1). The staining intensity increased in aged animals (*arrows*). (**B**) Central retina of adult and aged WT mice. *Scale bar*: 50 µm.

**Figure 4.**
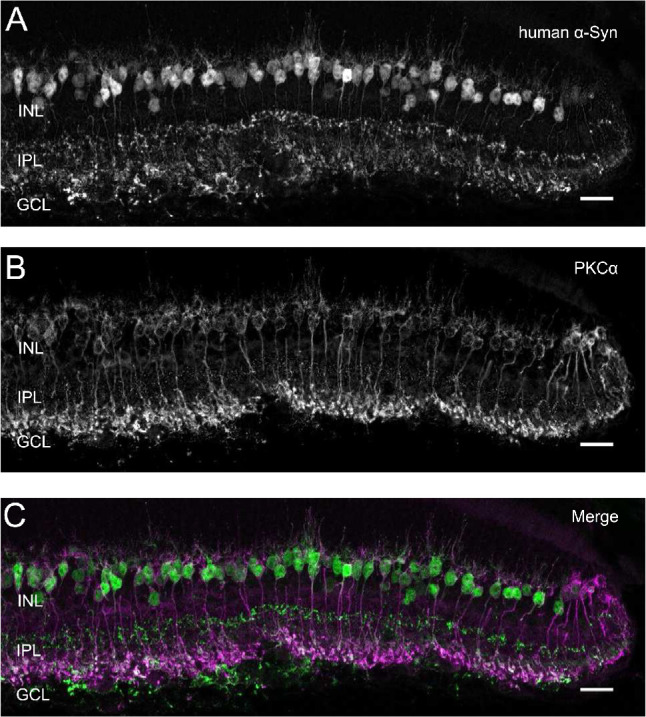
Human α-Syn immunoreactivity in rod bipolar cells. Co-staining of (**A**) human α-Syn and (**B**) PKCα in rod bipolar cells of Plp-α-Syn. (**C)** Merged image of human α-Syn and PKCα. The age of animals was 12 months. *Scale bar*: 20 µm.

Our immunohistochemical analyses discovered human α-Syn in the inner plexiform and ganglion cell layer in vertical sections of the central retina of Plp-α-Syn mice (Fig. 3A), but not in wild-type controls (Fig. 3B). In the peripheral retina, we detected human α-Syn in the inner nuclear layer (INL) (Fig. 3A). This effect was pronounced in aged animals (Fig. 3A, arrows). Due to the localization of the cell bodies adjacent to the outer plexiform layer, we suggested that these were rod bipolar cells. Co-staining human α-Syn (Fig. 4A, Supplementary Fig. S2) with the specific rod bipolar cell marker PKCα (Fig. 4B) supported this notion (Fig. 4C).

**Figure 5.**
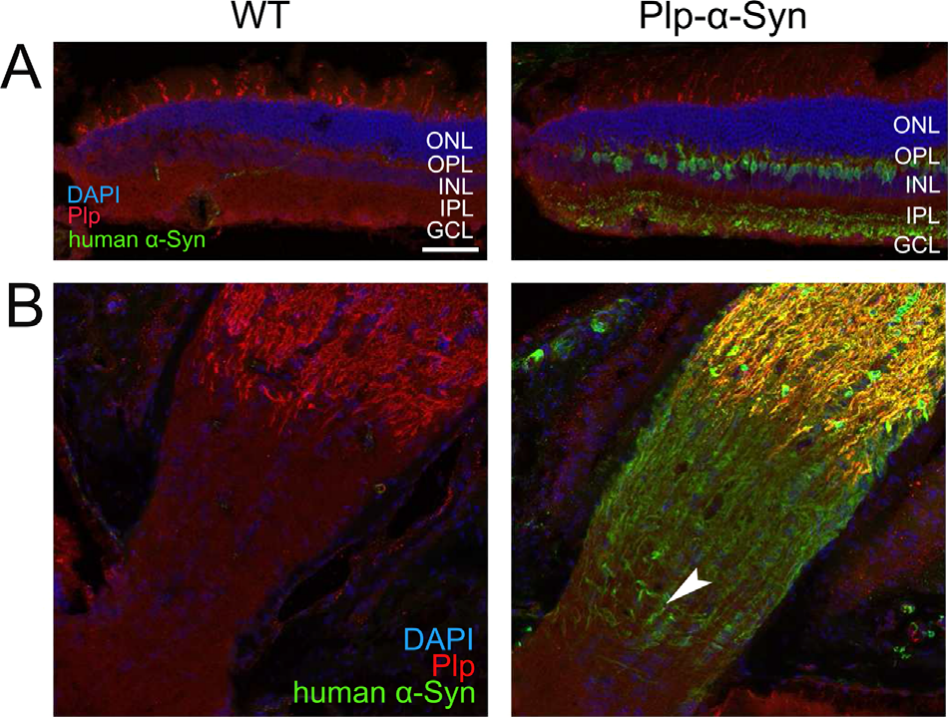
Plp in mouse retina and optic nerve. (**A**) Retinal sections stained with anti-Plp (*red*) and anti-human α-Syn (*green*) antibodies indicated that wild-type (WT) and Plp-α-Syn retinas are devoid of Plp protein. No colocalization occurred with human α-Syn in rod bipolar cells of Plp-α-Syn retinas (*right panel*). (**B**) Co-labeling of Plp and human α-Syn in the optic nerve of WT and Plp-α-Syn animals. Expression of human α-Syn proceeded toward the retina (*arrowhead*). Age of animals was 8 to 10 weeks. *Scale bar*: 50 µm.

In line with previous reports,^54^^,^^55^ we did not detect Plp immunoreactivity in the retinas of wild-type and Plp-α-Syn mice (Fig. 5A). In particular, we did not observe Plp protein in rod bipolar cells containing human α-Syn (Fig. 5A, right panel). In the optic nerve, which we used as a reference, Plp was more pronounced than in the retina. As expected, Plp stopped at the retina–optic nerve junction (Fig. 5B). In Plp-α-Syn mice, we detected human α-Syn in the optic nerve, where it colocalized with Plp, but also closer toward the retina (Fig. 5B, arrowhead). We measured colocalization between Plp and human α-Syn and compared two regions, where the optic nerve enters the retina compared to a more distal region on the optical nerve (Supplementary Fig. S3, Supplementary Table S1). These analyses showed the close spatial proximity of human α-Syn with respect to the Plp that oligodendrocytes in the distal optic nerve produce.

### Retinal Structure in Plp-α-Syn Mice

**Figure 6.**
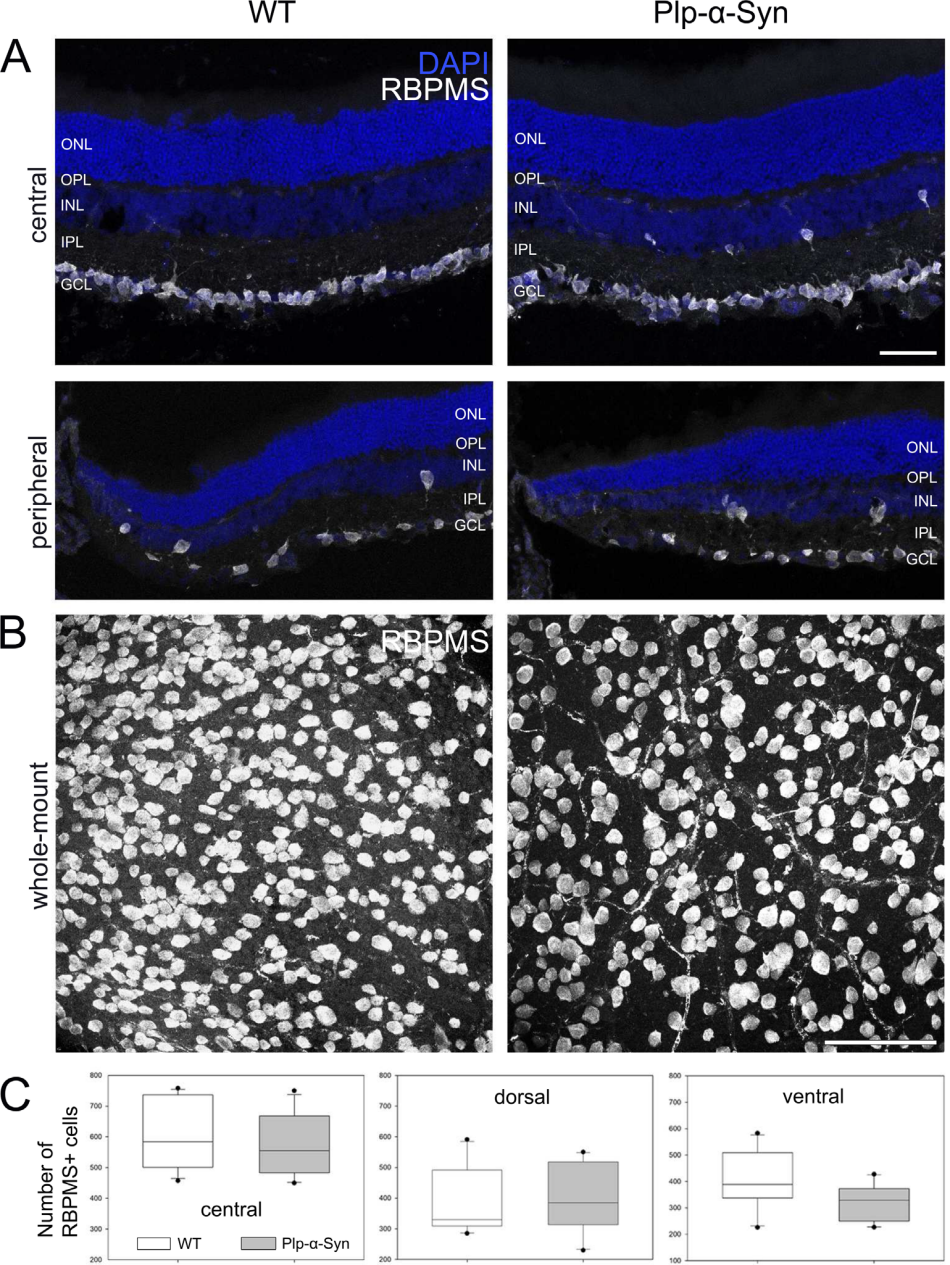
Retinal ganglion cells in adult retinas. (**A**) Immunolabeling with the global marker RNA-binding protein with multiple splicing (RBPMS) in central and peripheral sections from adult wild-type (WT) and Plp-α-Syn retinas. *Scale bar*: 50 µm. (**B**) Representative RBPMS staining in the ventral retinal whole-mount of WT and Plp-α-Syn mice. *Scale bar*: 100 µm. (**C**) In the ventral Plp-α-Syn retina, we observed a trend toward a reduction in the retinal ganglion cell number. *P* = 0.06, Student's *t*-test (N = 10). Data are shown as mean ± SEM. The age of animals was 8 to 10 weeks.

MSA patients show a reduction in ganglion cell density in the peripheral retina.^53^^,^^56^ We therefore also investigated retinal ganglion cells in sections and whole-mounts of adult Plp-α-Syn animals using the global marker RNA-binding protein with multiple splicing (Fig. 6). Whereas the ganglion cell shape seemed unaffected (Figs. 6A, 6B), we found a trend toward a reduction in their cell number (Fig. 6C, ventral) compared to wild-type retinas. Overall, the thickness of the different retinal layers was comparable in both adult and aged wild-type and Plp-α-Syn mice (Supplementary Fig. S4).

### Glial Cells in Retinas Overexpressing Human α-Syn

**Figure 7.**
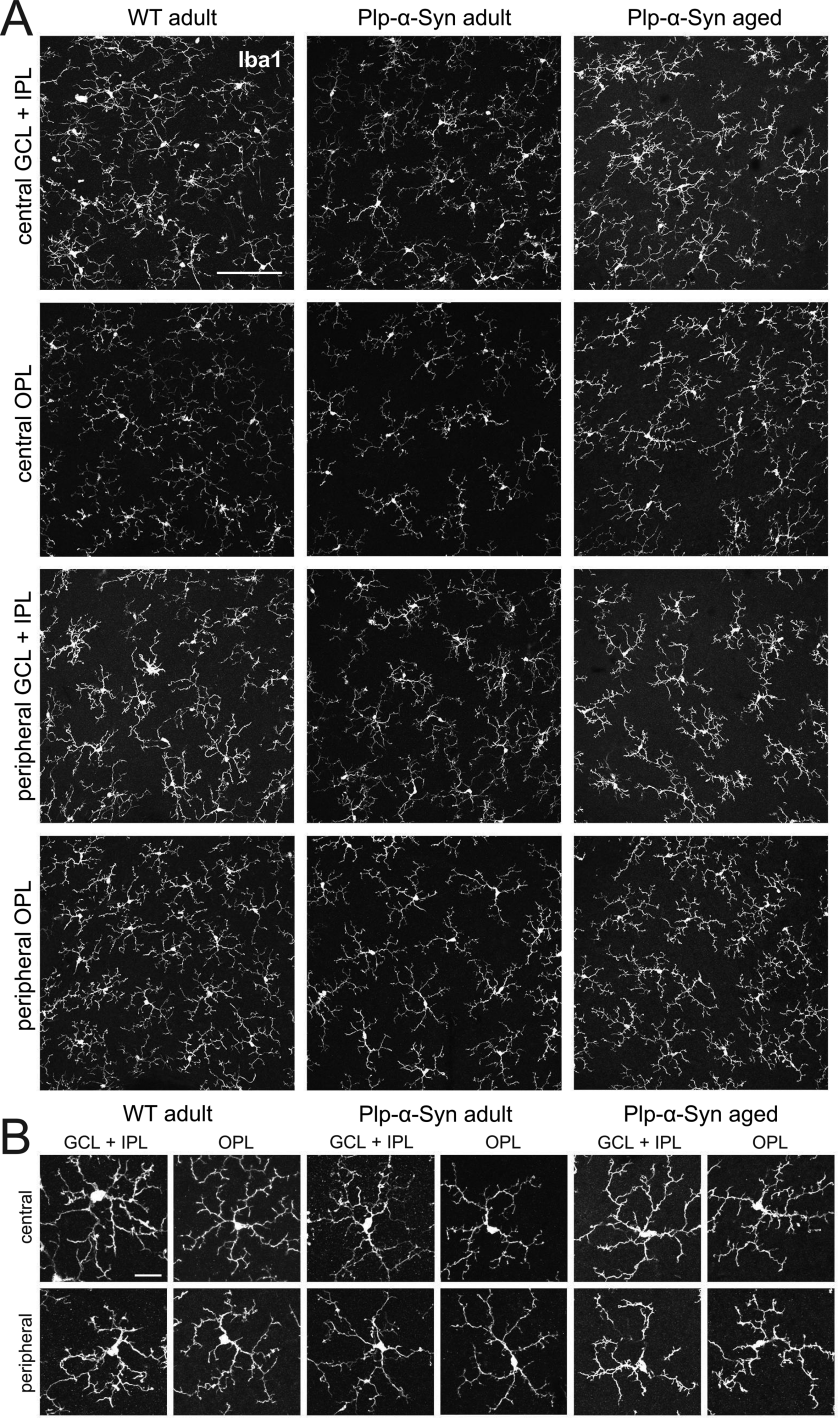
Retinal microglia in adult and aged retinas. Retinal whole-mounts from wild-type (WT) and Plp-α-Syn (adult, 8 weeks, and aged, 12 months) were stained with the microglia marker Iba1. **(A)** Collapsed *Z*-stack images show GCL + IPL and OPL of central and dorsal retinas. No microglia activation was detectable in Plp-α-Syn retinas. *Scale bar*: 200 µm. (**B**) Representative microglia cells in different retinal layers of central and peripheral parts of the retina. *Scale bar*: 10 µm.

In the brain of Plp-α-Syn mice,^57^^,^^58^ microglia activation was comparable to the human condition.^10^^,^^59^ We were therefore also interested in retinal microglia. To examine potential changes in the activation state of glial cells in the retina we performed immunostaining with the microglia marker Iba1^60^ and GFAP, reported to accumulate in response to retinal degeneration.^61^ We did not find evidence for activated microglia in the ganglion cell and the outer plexiform layer of whole-mounted retinas (Fig. 7A); rather, microglia appeared in a homeostatic state characterized by a small cell body and long ramified processes (Fig. 7B). Quantitative analyses of Iba1^+^ microglia in Plp-α-Syn retinas revealed that their processes covered a smaller area in the outer plexiform layer but not in the ganglion cell layer compared to wild-type retinas (Supplementary Fig. S5A). We also found reduced microglia cell body areas (Supplementary Fig. S5B) which we could not explain by a reduction in the size of the single cell bodies (Supplementary Fig. S5C) but rather by a reduction of Iba1^+^ microglia cell numbers (Supplementary Fig. S5D).

**Figure 8.**
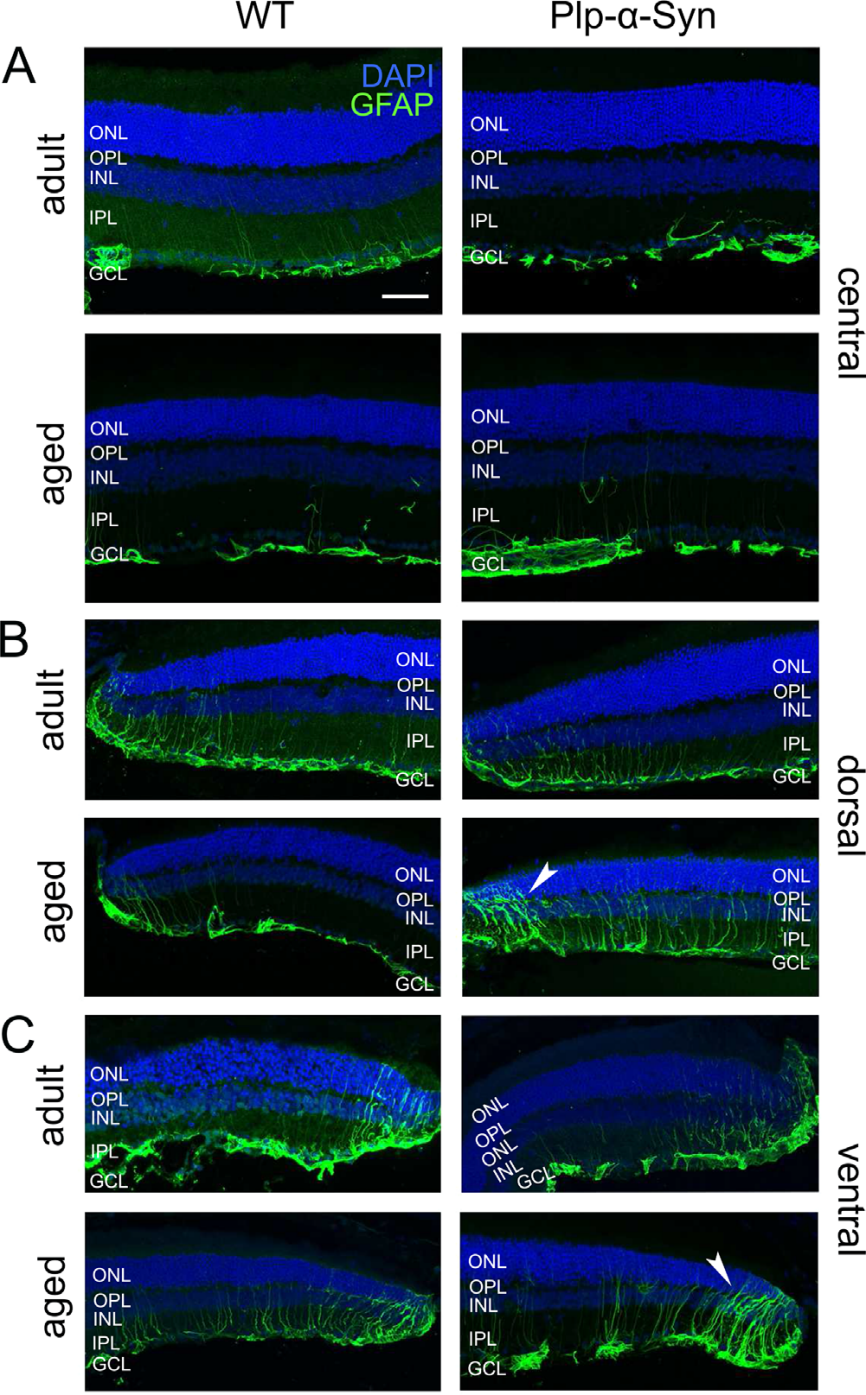
Immunoreactivity against glial fibrillary acidic protein. GFAP (*green*) in the central (**A**) and peripheral (**B, C**) retinas of adult (8 to 10 weeks) and aged (12 months) wild-type (WT) and Plp-α-Syn mice. Activated Müller glia cells were also seen in WT peripheral retinas (**B**, **C**, left panel). In aged Plp-α-Syn retinas, GFAP accumulation reached the ONL (*arrowheads*). *Scale bar*: 50 µm.

The anti-GFAP stainings in Figure 8 show that in adult central retinas GFAP was mainly concentrated in the end feet region of Müller cells and/or astrocytes, whereas in the periphery GFAP expanded into the ONL (Figs. 8B, 8C). In aged mice, GFAP was present in the inner and outer nuclear layers (Figs. 8B, 8C, arrowheads), and the increase in GFAP accumulation seemed to proceed from the central to the peripheral retina, where it reached into the ONL. Quantification of GFAP-positive areas showed differences between central and peripheral areas of the retina in adult (*P* = 0.0009, unpaired *t*-test) and aged animals (*P* = 0.0087, unpaired *t*-test). GFAP-positive signals were similar in retinas of wild-type and Plp-α-Syn mice, with the following %ROI values: For adult wild-type retinas, center = 3.91 ± 0.80 and periphery = 9.88 ± 2.47; for aged wild-type retinas, center = 2.97 ± 1.09 and periphery = 8.15 ± 1.53. For adult Plp-α-Syn mice, center = 3.73 ± 0.96 and periphery = 9.43 ± 0.86; for aged Plp-α-Syn mice, center = 3.47 ± 0.57 and periphery = 8.90 ± 3.04. Data are presented as mean ± SEM from three animals for each group.

### Dopaminergic Retinal Neurons in the Plp-α-Syn Mice

**Figure 9.**
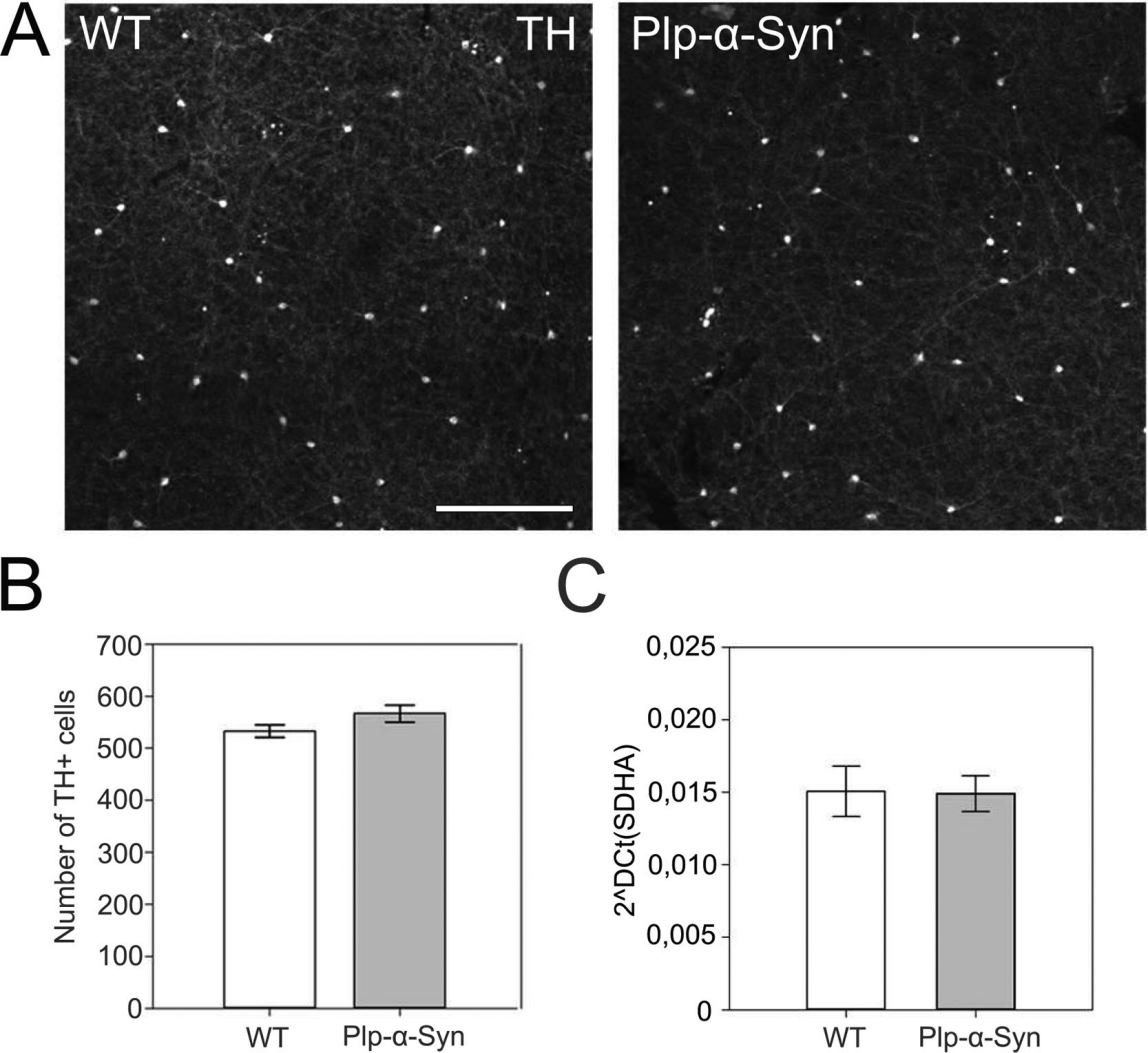
Tyrosine hydroxylase immunoreactive neurons. (**A**) Retinal whole-mount staining for TH. *Scale bar*: 100 µm. (**B**) The total numbers of dopaminergic neurons were comparable (wild-type: 533 ± 30 cells, N = 6; Plp-α-Syn: 567 ± 46 cells; data are given as mean ± SEM; N = 8 for both groups). The age of animals was 8 to 10 weeks. *P* = 0.12, Student's *t*-test. (**C**) TH mRNA expression levels did not differ between wild-type and Plp-α-Syn: Ct values were normalized to the housekeeping gene *Sdha*: WT: 0.015 ± 0.0038, N = 5; Plp-α-Syn: 0.015 ± 0.0027, N = 5. *P* = 0.94, Student's *t*-test.

In synucleinopathies such as PD, the dopaminergic system has been shown to be severely impacted.^62^ In the retina, dopaminergic amacrine cells are interneurons that modulate key visual processes, such as light adaptation, but they also have trophic roles in retinal function.^63^ To investigate dopaminergic retinal neurons in Plp-α-Syn mice we performed tyrosine hydroxylase (TH) stainings in whole-mounted retinas. Quantification of TH+ cells showed no change in the number of cells in Plp-α-Syn retinas as compared to wild-type retinas (Figs. 9A, 9B). In line with this observation, TH mRNA levels also did not differ between wild-type and Plp-α-Syn retinas (Fig. 9C).

### Functional Activity of Plp-α-Syn Retinas

**Figure 10.**
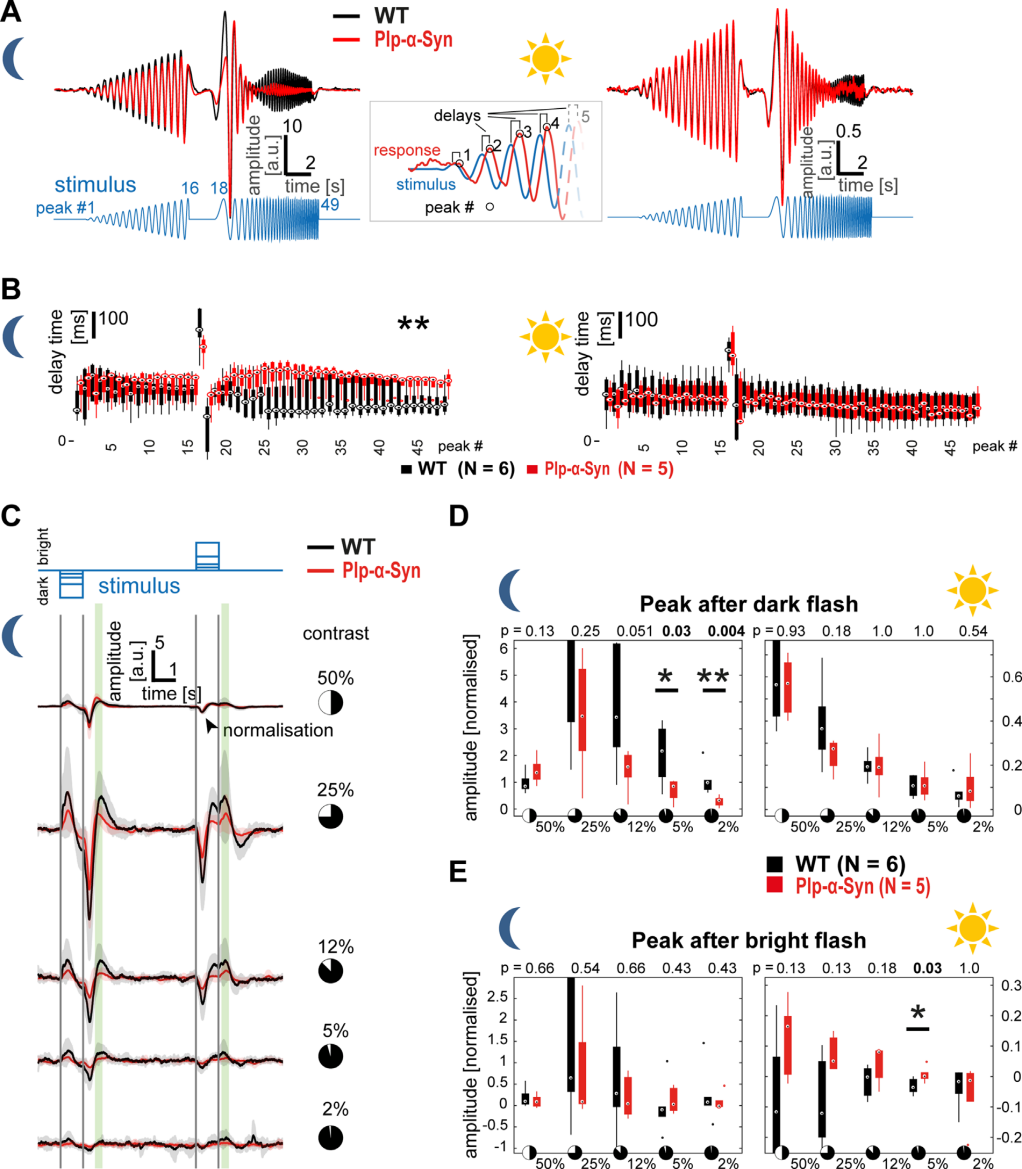
Microelectrode array-based ex vivo ERG recordings from retinal whole-mounts. (**A**) Traces show average responses of wild-type (WT, *black*, N = 6) and Plp-α-Syn (*red*, N = 5) retinas from animals 8 to 11 weeks old in scotopic (left) and photopic (right) luminance. The stimulus was a full-field contrast- and frequency-modulated sinusoidal (chirp). Response peak delays (inset) were quantified (peak numbers: 1–16, contrast-modulated; 17, end of contrast modulation; 18–49, frequency modulated). (**B**) In scotopic luminance (left), delay times were longer in Plp-α-Syn retinas in the frequency-modulated part. ^*^P < 0.05, Wilcoxon rank-sum test. In photopic luminance (right), no difference was observed. In the box plots, *circles* represent medians, *boxes* are the 25th to 75th percentiles, and *whiskers* are the most extreme data points. (**C**) Responses to a full-field flash stimulus consisting of negative contrasts (dark flashes) and positive contrasts (bright flashes) in scotopic luminance. Traces show average responses of WT (*black*, N = 6) and Plp-α-Syn (*red*, N = 5) retinas to five different contrast levels (50% to 2% Weber contrast); shaded areas represent the SD. Maximum responses (largest positive deflections) at two distinct time points (*green shading*) were analyzed. (**D**) Maximal responses after the dark flash showed differences for the two lowest contrasts in scotopic luminance whereas the bright flash revealed only a single change at 5% contrast in photopic luminance. ^*^P < 0.05, ^**^P < 0.01, Wilcoxon rank-sum test. In the box plots, *boxes* are the 25th to 75th percentiles, *whiskers* are the most extreme data points, and *dots* are outliers.

**Figure 11.**
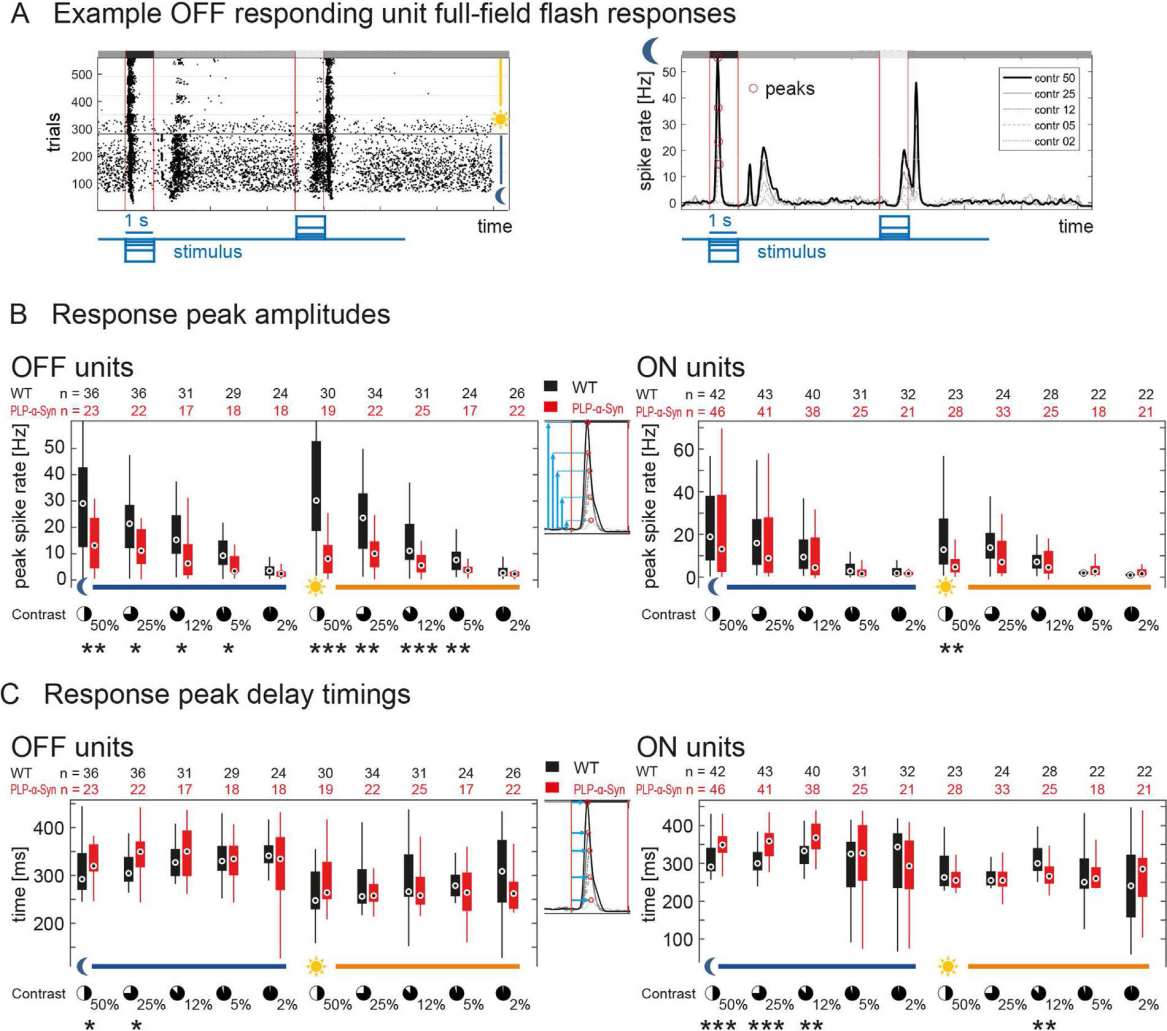
Ganglion cell spiking activity recorded on MEAs. (**A**) Example spiking responses of a wild-type OFF ganglion cell to the full-field flash stimulus. (*Left*) Raster plot of all trials; each dot represents a spike, luminance level switch is indicated. (*Right*) Average convolved spike rates to all flash contrasts in scotopic luminance. (**B**) Peak amplitudes of preferred polarity flash responses of OFF (*left*) and ON (*right*) ganglion cells across all contrasts in both luminance levels. Amplitudes were significantly different for higher contrasts in both luminance levels (OFF cells) and at 50% contrast in photopic luminance (ON cells). (**C**) Peak delay timings of preferred polarity flash responses of OFF (left) and ON (right) ganglion cells across all contrasts in both luminance levels. Delays were significantly different, mainly in scotopic luminance at higher contrasts for both OFF and ON cells. ^*^P < 0.05, ^**^P < 0.01, ^***^P < 0.001, Wilcoxon rank-sum test. *Circles* represent medians, *boxes* are the 25th to 75th percentiles, and *whiskers* are the most extreme data points. The numbers differ because individual cell responses could not be measured at all contrasts. The age of animals was 8 to 11 weeks.

To test the impact of human α-Syn accumulation on the function of the retina, we performed MEA recordings in adult wild-type and Plp-α-Syn retinal whole-mounts. We extracted the local field potential, a slow-wave potential regarded as a form of ex vivo ERG that mainly reveals photoreceptor and bipolar cell activation upon visual stimulation. First, we investigated responses to a full-field “chirp” stimulus that provided insight into aspects of sensitivity and timing of outer retina function in both scotopic (rod-driven) and photopic (mostly cone-driven) conditions (Fig. 10A). We found a significant delay in the responses to the frequency-modulated part of the chirp only at higher frequencies (>5 Hz) and specifically under scotopic conditions (Fig. 10B, left). Likewise, we observed changes in the response amplitudes only in scotopic luminance (data not shown). Within the photopic regime, neither timing nor amplitude of the responses was different between wild-type and Plp-α-Syn retinas (Fig. 10B, right). In a second step, this observation was confirmed with a full-field flash stimulus (Fig. 10C) consisting of 1-second steps of different negative and positive contrasts (±2% to ±50% Weber contrast). We found reduced response amplitudes mainly within scotopic luminance. Response amplitude changes were restricted to the lowest flash contrasts, specifically to the 5% and 2% contrast flashes (Fig. 10D). In photopic luminance, only a single significant difference in the response to the positive contrast flash was observed (Fig. 10E). We also investigated retina function on the output level and analyzed spiking responses of retinal ganglion cells to the full-field flashes, based on the same MEA recordings used for the ERG. Figure 11A shows representative responses of a wild-type OFF cell to all trials and flash contrasts across both luminance levels (raster plot, left) and the average responses to different contrast flashes in scotopic luminance (mean convolved spike rates, right). The peak amplitudes to most contrasts were significantly reduced in OFF responding units of Plp-α-Syn retinas, in both scotopic and photopic luminance levels (Fig. 11B, left). ON responding units had similar response amplitudes in both genotypes, except for the 50% contrast flash in photopic luminance (Fig. 11B, right). Peak delay timings were significantly increased in Plp-α-Syn ganglion cells for both OFF and ON responding units, mainly in the scotopic luminance level (Fig. 11C). Together our functional data indicate that the mild differences in outer and inner retinal function were mostly at the scotopic luminance level, whereas photopic function seemed less altered.

## Discussion

Due to a variety of visual symptoms observed in patients with synucleinopathies, a role for the neuroretina as a biomarker for disease progression and/or differential diagnosis is being discussed. Evidence has been provided by optical coherence tomography (OCT) studies,^33^^,^^53^^,^^64^^,^^65^ as well as morphological investigations in human tissue.^34^^,^^35^^,^^56^^,^^66^^,^^67^ Studies consistently indicated a disease-induced retinal impairment in MSA patients. Because of the accessibility of the retina for such non-invasive techniques as OCT, which allows the ophthalmologist to map and measure the distinctive layers of the retina, and because of the urgent need for an early diagnostic marker to ease the disease burden of patients, investigating the retina in MSA mouse models is important.

### Neuroretina in Plp-α-Syn Mice

Due to the nature of the Plp-α-Syn MSA model used in this study, accumulation of human α-Syn in the optic nerve was expected. Plp, the major myelin protein (OMIM 300112^4^) and the promoter driving α-Syn expression in the present model, is expressed in oligodendrocytes,^68^ which are absent from the retina but abundant in the optic nerve. Effects on retinal ganglion cells were anticipated, because the axonal/oligodendroglia pathology can lead to ganglion cell death.^69^^–^^72^ In our study, we observed a trend toward a decrease in the ganglion cell density in the ventral part of the adult Plp retina.

Although the retina is devoid of oligodendroglia cells and Plp protein,^54^^,^^55^ we observed human α-Syn protein in the retina. Our western blot experiments supported the presence of human α-Syn in the retina. We also found phosphorylated α-Syn in both wild-type and Plp-α-Syn retinas. Phosphorylation of α-Syn, however, is not necessarily only pathological^73^ but also has implications in the regulation of physiological functions (for a review, see Oueslati^73^). We therefore suggest that the levels observed represent baseline physiological phosphorylation levels.

Notably human α-Syn was visible in rod bipolar cells. This pronounced accumulation of α-Syn in rod bipolar cells is unique and to the best of our current knowledge not yet described in any other synucleinopathy model. Comparable to our observations, human microtubule-associated protein Tau in an Alzheimer's disease mouse model accumulated in rod bipolar cells in the inner retina. Tau was also located in the axonal compartment of retinal ganglion cells; however, light-induced retinal cell activation was not altered.^74^

In the Plp-α-Syn model, along with the more pronounced expression of human α-Syn protein in rod bipolar cells of the peripheral retina, GFAP-accumulating Müller cells also increased from the center to the periphery. The increase of this established marker of retinal stress indicated stronger degenerative effects in the retinal periphery reminiscent of human patient data.^56^ However, we also found GFAP accumulation in peripheral wild-type retinas. This finding in wild-type retinas is difficult to compare with published literature because most studies focus on the central retina only, and this observation is most likely not related to retinal reactivity. With respect to microglia activation, our findings in the retina differ from those for the brain, where region-specific, α-Syn-associated microglia activation has been reported.^58^ We found no evidence for activated microglia; type A or A/B microglia corresponding to surveillant/homeostatic microglia were presented but not type C (hypertrophic) or D (amoeboid).^58^ In the current study, our semiquantitative analyses of the microglia pool in the outer plexiform layer showed a tendency toward a reduction in the Plp-α-Syn retinas with a reduced area covered by microglia processes; however, we cannot conclude that we see a clear loss of microglia cells. Further studies will have to be undertaken to determine the underlying causes. Of note, microglia depletion in mice did not result in changes of the general retinal architecture, organization of second-order neurons, or photoreceptor structure but did lead to decreased ability of the retina to transmit light.^75^

In our mouse model, we cannot fully exclude that the observed accumulation is due to ectopic expression of the Plp-α-Syn construct, because α-Syn could accumulate over a longer period even if mRNA transcript levels were low. Still, our findings implicate that retinal bipolar neurons can cope with increased levels of α-Syn protein because its expression did not cause any further morphological changes, and functionality was not strongly impacted as was seen in our MEA recordings.

α-Syn-expressing retinal neurons might also lack the appropriate clearing mechanisms to reduce the α-Syn load because intracellular homeostasis of α-Syn requires proper degradation of the protein. Different mechanisms (e.g., chaperone-mediated autophagy, macroautophagy, ubiquitin–proteasome system) have been demonstrated to be involved in α-Syn degradation.^76^^–^^80^ We therefore propose the use of this Plp-α-Syn mouse model to further study the mechanisms of α-Syn aggregation and its clearance pathways.

### Functionality of the Plp-α-Syn Retina

Despite exhibiting substantial accumulation of α-Syn in the inner retina and ganglion cells and the (peripheral) rod bipolar cells in particular, we found little change in retinal function of Plp-α-Syn mice. The changes in outer retina function we observed in our ex vivo ERG data were largely restricted to scotopic luminance levels, suggesting an involvement of rod-driven retinal circuitry. This finding correlates with the presence of α-Syn in rod bipolar cells, which are crucial for a major part of rod-derived signal transfer down to the inner retina. Even though α-Syn labeling of rod bipolar cells was most intense in the periphery, we saw no difference in the response profiles between the central and most peripheral parts of the retina (data not shown); therefore, we conclude that the levels of α-Syn present in these neurons at most mildly affect their function. Of note, we observed differences in ex vivo ERG elicited by stimuli with the weakest driving force for responses: high frequencies (in the chirp) and low contrasts (in the flash). These findings suggest an impairment of outer retinal signaling at the outskirts of the normal dynamic range, whereas stronger stimulation reveals no difference in ex vivo ERG. Ganglion cell responses by comparison differed more at higher contrast levels, reflecting either an additional inner retina effect or a result from an altered balance in synaptic gains driving these responses.

Because ERG is a non-invasive routine diagnostic tool in clinical application, the ex vivo ERG phenotype at low contrasts is interesting. Previous studies with patients failed to reveal substantial visual phenotypes.^40^^,^^81^^–^^83^ These studies, however, regularly used stimulation above cone threshold or higher contrast stimuli. Therefore, with regard to patient diagnosis, it might be worth measuring visual performance in ERGs with higher temporal frequencies and very low-contrast stimuli, specifically at scotopic luminance, as inferred by our MEA analyses.

### Dopaminergic Neurons in the Plp-α-Syn Retina

Whereas cerebral loss of dopaminergic neurons has been shown in the substantia nigra of Plp-α-Syn mice,^58^ data on retinal dopaminergic neurons have not yet been available for this model. Similar to our study, where we demonstrated comparable numbers of TH+ neurons and comparable levels of TH mRNA, a recent report in transgenic A53T mice also showed no changes in dopaminergic amacrine cells in retinal sections.^84^ We therefore concluded that the retina of the Plp-α-Syn model does not show an impairment of the dopaminergic system, a finding that compares to observations in the olfactory bulb of Plp-α-Syn mice.^85^

### How Does the Effect on the Ganglion Cell Morphology Reflect the Human Situation in MSA Patients?

Retinal ganglion cell density has been shown to be significantly reduced in the peripheral retina of MSA patients.^56^ Even though the difference in the number of ganglion cells did not reach significance in the Plp-α-Syn mouse model, it seemed decreased in the ventral retina. A possible cause for an impairment could be differences in myelination, as suggested for the peripheral human retina that contains M-type retinal ganglion cells. Their long axons show a high level of myelination in the retrolaminar optic nerve leading to a higher vulnerability to disease-prone oligodendrocyte damage.^64^ Studies examining axonal myelination and degeneration in this mouse model exceeded the scope of this study. The fact that we found human α-Syn expressed in the retrolaminar optic nerve co-localizing with Plp but also towards the retina makes this an interesting follow up question.

Taken together, we found the MSA pathology partly reflected in the Plp-α-Syn mouse model, although the morphological and functional changes might be too subtle for a biomarker. However, due to the observed α-Syn expression in retinal neurons, Plp-α-Syn mice could serve as a useful biological model to study synuclein-dependent diseases in the retina, a uniquely accessible neuronal tissue.

## Supplementary Material

Supplement 1

Supplement 2

Supplement 3

Supplement 4

Supplement 5

Supplement 6

## References

[1] Stefanova N, Bucke P, Duerr S, Wenning GK Multiple system atrophy: an update. *Lancet Neurol*. 2009; 8: 1172–1178. 19909915 10.1016/S1474-4422(09)70288-1

[2] Fanciulli A, Wenning GK Multiple-system atrophy. *N Engl J Med*. 2015; 372: 1375–1376. 10.1056/NEJMc1501657 25830435

[3] Wenning GK, Ben Shlomo Y, Magalhaes M, Daniel SE, Quinn NP Clinical features and natural history of multiple system atrophy. An analysis of 100 cases. *Brain*. 1994; 117: 835–845. 7922469 10.1093/brain/117.4.835

[4] Amberger JS, Bocchini CA, Schiettecatte F, Scott AF, Hamosh A OMIM.org: Online Mendelian Inheritance in Man (OMIM(R)), an online catalog of human genes and genetic disorders. *Nucleic Acids Res*. 2015; 43: D789–D798. 25428349 10.1093/nar/gku1205 PMC4383985

[5] Bellani S, Sousa VL, Ronzitti G, Valtorta F, Meldolesi J, Chieregatti E The regulation of synaptic function by alpha-synuclein. *Commun Integr Biol*. 2010; 3: 106–109. 20585500 10.4161/cib.3.2.10964 PMC2889964

[6] Lim S, Chun Y, Lee JS, Lee SJ Neuroinflammation in synucleinopathies. *Brain Pathol*. 2016; 26: 404–409. 26940152 10.1111/bpa.12371 PMC8028946

[7] Taguchi K, Watanabe Y, Tsujimura A, et al. Differential expression of alpha-synuclein in hippocampal neurons. *PLoS One*. 2014; 9: e89327. 24586691 10.1371/journal.pone.0089327 PMC3934906

[8] Burre J, Sharma M, Sudhof TC Cell biology and pathophysiology of alpha-synuclein. *Cold Spring Harb Perspect Med*. 2018; 8: a024091. 28108534 10.1101/cshperspect.a024091 PMC5519445

[9] McCann H, Stevens CH, Cartwright H, Halliday GM alpha-Synucleinopathy phenotypes. *Parkinsonism Relat Disord*. 2014; 20(suppl 1): S62–S67. 24262191 10.1016/S1353-8020(13)70017-8

[10] Papp MI, Kahn JE, Lantos PL Glial cytoplasmic inclusions in the CNS of patients with multiple system atrophy (striatonigral degeneration, olivopontocerebellar atrophy and Shy-Drager syndrome). *J Neurol Sci*. 1989; 94: 79–100. 2559165 10.1016/0022-510x(89)90219-0

[11] Spillantini MG Parkinson's disease, dementia with Lewy bodies and multiple system atrophy are alpha-synucleinopathies. *Parkinsonism Relat Disord*. 1999; 5: 157–162. 18591134 10.1016/s1353-8020(99)00031-0

[12] Wakabayashi K, Yoshimoto M, Tsuji S, Takahashi H Alpha-synuclein immunoreactivity in glial cytoplasmic inclusions in multiple system atrophy. *Neurosci Lett*. 1998; 249: 180–182. 9682846 10.1016/s0304-3940(98)00407-8

[13] Tu PH, Galvin JE, Baba M, et al. Glial cytoplasmic inclusions in white matter oligodendrocytes of multiple system atrophy brains contain insoluble alpha-synuclein. *Ann Neurol*. 1998; 44: 415–422. 9749615 10.1002/ana.410440324

[14] Spillantini MG, Crowther RA, Jakes R, Cairns NJ, Lantos PL, Goedert M Filamentous alpha-synuclein inclusions link multiple system atrophy with Parkinson's disease and dementia with Lewy bodies. *Neurosci Lett*. 1998; 251: 205–208. 9726379 10.1016/s0304-3940(98)00504-7

[15] Papp MI, Lantos PL Accumulation of tubular structures in oligodendroglial and neuronal cells as the basic alteration in multiple system atrophy. *J Neurol Sci*. 1992; 107: 172–182. 1314292 10.1016/0022-510x(92)90286-t

[16] Michel PP, Hirsch EC, Hunot S Understanding dopaminergic cell death pathways in Parkinson disease. *Neuron*. 2016; 90: 675–691. 27196972 10.1016/j.neuron.2016.03.038

[17] Cykowski MD, Coon EA, Powell SZ, et al. Expanding the spectrum of neuronal pathology in multiple system atrophy. *Brain*. 2015; 138: 2293–2309. 25981961 10.1093/brain/awv114 PMC4840945

[18] Shults CW, Rockenstein E, Crews L, et al. Neurological and neurodegenerative alterations in a transgenic mouse model expressing human alpha-synuclein under oligodendrocyte promoter: implications for multiple system atrophy. *J Neurosci*. 2005; 25: 10689–10699. 16291942 10.1523/JNEUROSCI.3527-05.2005 PMC6725840

[19] Rockenstein E, Ubhi K, Inglis C, et al. Neuronal to oligodendroglial alpha-synuclein redistribution in a double transgenic model of multiple system atrophy. *NeuroReport*. 2012; 23: 259–264. 22314685 10.1097/WNR.0b013e3283509842 PMC3289254

[20] Wakabayashi K, Takahashi H Cellular pathology in multiple system atrophy. *Neuropathology*. 2006; 26: 338–345. 16961071 10.1111/j.1440-1789.2006.00713.x

[21] Jellinger KA, Lantos PL Papp-Lantos inclusions and the pathogenesis of multiple system atrophy: an update. *Acta Neuropathol*. 2010; 119: 657–667. 20309568 10.1007/s00401-010-0672-3

[22] Goedert M, Jakes R, Spillantini MG The synucleinopathies: twenty years on. *J Parkinsons Dis*. 2017; 7(suppl 1): S51–S69. 28282814 10.3233/JPD-179005 PMC5345650

[23] Kahle PJ, Neumann M, Ozmen L, et al. Hyperphosphorylation and insolubility of alpha-synuclein in transgenic mouse oligodendrocytes. *EMBO Rep*. 2002; 3: 583–588. 12034752 10.1093/embo-reports/kvf109 PMC1084143

[24] Fellner L, Wenning GK, Stefanova N Models of multiple system atrophy. *Curr Top Behav Neurosci*. 2015; 22: 369–393. 24338664 10.1007/7854_2013_269 PMC4730554

[25] Stemberger S, Poewe W, Wenning GK, Stefanova N Targeted overexpression of human alpha-synuclein in oligodendroglia induces lesions linked to MSA-like progressive autonomic failure. *Exp Neurol*. 2010; 224: 459–464. 20493840 10.1016/j.expneurol.2010.05.008 PMC2913120

[26] Kuzdas D, Stemberger S, Gaburro S, Stefanova N, Singewald N, Wenning GK Oligodendroglial alpha-synucleinopathy and MSA-like cardiovascular autonomic failure: experimental evidence. *Exp Neurol*. 2013; 247: 531–536. 23399889 10.1016/j.expneurol.2013.02.002 PMC3748345

[27] Boudes M, Uvin P, Pinto S, et al. Bladder dysfunction in a transgenic mouse model of multiple system atrophy. *Mov Disord*. 2013; 28: 347–355. 23426727 10.1002/mds.25336 PMC4743066

[28] Hartner L, Keil TW, Kreuzer M, et al. Distinct parameters in the EEG of the PLP alpha-SYN mouse model for multiple system atrophy reinforce face validity. *Front Behav Neurosci*. 2016; 10: 252. 28119583 10.3389/fnbeh.2016.00252 PMC5222844

[29] Stefanova N, Wenning GK Review: multiple system atrophy: emerging targets for interventional therapies. *Neuropathol Appl Neurobiol*. 2016; 42: 20–32. 26785838 10.1111/nan.12304 PMC4788141

[30] London A, Benhar I, Schwartz M The retina as a window to the brain-from eye research to CNS disorders. *Nat Rev Neurol*. 2013; 9: 44–53. 23165340 10.1038/nrneurol.2012.227

[31] Fischer MD, Synofzik M, Kernstock C, et al. Decreased retinal sensitivity and loss of retinal nerve fibers in multiple system atrophy. *Graefes Arch Clin Exp Ophthalmol*. 2013; 251: 235–241. 22878471 10.1007/s00417-012-2118-1

[32] Ahn J, Lee JY, Kim TW Retinal thinning correlates with clinical severity in multiple system atrophy. *J Neurol*. 2016; 263: 2039–2047. 27416856 10.1007/s00415-016-8230-0

[33] Chorostecki J, Seraji-Bozorgzad N, Shah A, et al. Characterization of retinal architecture in Parkinson's disease. *J Neurol Sci*. 2015; 355: 44–48. 26071887 10.1016/j.jns.2015.05.007

[34] Bodis-Wollner I, Kozlowski PB, Glazman S, Miri S alpha-Synuclein in the inner retina in Parkinson disease. *Ann Neurol*. 2014; 75: 964–966. 24816946 10.1002/ana.24182

[35] Beach TG, Carew J, Serrano G, et al. Phosphorylated alpha-synuclein-immunoreactive retinal neuronal elements in Parkinson's disease subjects. *Neurosci Lett*. 2014; 571: 34–38. 24785101 10.1016/j.neulet.2014.04.027 PMC4591751

[36] Leger F, Fernagut PO, Canron MH, et al. Protein aggregation in the aging retina. *J Neuropathol Exp Neurol*. 2011; 70: 63–68. 21157377 10.1097/NEN.0b013e31820376cc

[37] Bodis-Wollner I, Marx MS, Mitra S, Bobak P, Mylin L, Yahr M Visual dysfunction in Parkinson's disease. Loss in spatiotemporal contrast sensitivity. *Brain*. 1987; 110: 1675–1698. 3427405 10.1093/brain/110.6.1675

[38] Armstrong RA Visual symptoms in Parkinson's disease. *Parkinsons Dis*. 2011; 2011: 908306. 21687773 10.4061/2011/908306 PMC3109513

[39] Regan D, Maxner C Orientation-selective visual loss in patients with Parkinson's disease. *Brain*. 1987; 110: 415–432. 3567530 10.1093/brain/110.2.415

[40] Delalande I, Hache JC, Forzy G, Bughin M, Benhadjali J, Destee A Do visual-evoked potentials and spatiotemporal contrast sensitivity help to distinguish idiopathic Parkinson's disease and multiple system atrophy? *Mov Disord*. 1998; 13: 446–452. 9613735 10.1002/mds.870130312

[41] Tebartz van Elst L, Greenlee MW, Foley JM, Lucking CH Contrast detection, discrimination and adaptation in patients with Parkinson's disease and multiple system atrophy. *Brain*. 1997; 120: 2219–2228. 9448577 10.1093/brain/120.12.2219

[42] Sturm E, Fellner L, Krismer F, Poewe W, Wenning GK, Stefanova N Neuroprotection by epigenetic modulation in a transgenic model of multiple system atrophy. *Neurotherapeutics*. 2016; 13: 871–879. 27259295 10.1007/s13311-016-0447-1 PMC5081120

[43] Knoflach D, Kerov V, Sartori SB, et al. Cav1.4 IT mouse as model for vision impairment in human congenital stationary night blindness type 2. *Channels (Austin)*. 2013; 7: 503–513. 24051672 10.4161/chan.26368 PMC4042485

[44] Berg S, Kutra D, Kroeger T, et al. ilastik: interactive machine learning for (bio)image analysis. *Nat Methods*. 2019; 16: 1226–1232. 31570887 10.1038/s41592-019-0582-9

[45] Reinhard K, Tikidji-Hamburyan A, Seitter H, et al. Step-by-step instructions for retina recordings with perforated multi electrode arrays. *PLoS One*. 2014; 9: e106148. 25165854 10.1371/journal.pone.0106148 PMC4148441

[46] Heikkinen H, Vinberg F, Pitkanen M, Kommonen B, Koskelainen A Flash responses of mouse rod photoreceptors in the isolated retina and corneal electroretinogram: comparison of gain and kinetics. *Invest Ophthalmol Vis Sci*. 2012; 53: 5653–5664. 22743325 10.1167/iovs.12-9678

[47] Vinberg F, Kolesnikov AV, Kefalov VJ Ex vivo ERG analysis of photoreceptors using an in vivo ERG system. *Vision Res*. 2014; 101: 108–117. 24959652 10.1016/j.visres.2014.06.003 PMC4149224

[48] Tikidji-Hamburyan A, Reinhard K, Seitter H, et al. Retinal output changes qualitatively with every change in ambient illuminance. *Nat Neurosci*. 2015; 18: 66–74. 25485757 10.1038/nn.3891 PMC4338531

[49] Farrow K, Masland RH Physiological clustering of visual channels in the mouse retina. *J Neurophysiol*. 2011; 105: 1516–1530. 21273316 10.1152/jn.00331.2010 PMC3075295

[50] Schlick B, Flucher BE, Obermair GJ Voltage-activated calcium channel expression profiles in mouse brain and cultured hippocampal neurons. *Neuroscience*. 2010; 167: 786–798. 20188150 10.1016/j.neuroscience.2010.02.037 PMC3315124

[51] Pichler M, Cassidy TN, Reimer D, et al. β subunit heterogeneity in neuronal L-type Ca^2+^ channels. *J Biol Chem*. 1997; 272: 13877–13882. 9153247 10.1074/jbc.272.21.13877

[52] Burtscher V, Schicker K, Novikova E, et al. Spectrum of Cav1.4 dysfunction in congenital stationary night blindness type 2. *Biochim Biophys Acta*. 2014; 1838: 2053–2065. 24796500 10.1016/j.bbamem.2014.04.023 PMC4065569

[53] Mendoza-Santiesteban CE, Gabilondo I, Palma JA, Norcliffe-Kaufmann L, Kaufmann H The retina in multiple system atrophy: systematic review and meta-analysis. *Front Neurol*. 2017; 8: 206. 28596752 10.3389/fneur.2017.00206 PMC5443142

[54] Gao L, Macklin W, Gerson J, Miller RH Intrinsic and extrinsic inhibition of oligodendrocyte development by rat retina. *Dev Biol*. 2006; 290: 277–286. 16388796 10.1016/j.ydbio.2005.11.007

[55] Hovhannisyan A, Benkner B, Biesemeier A, Schraermeyer U, Kukley M, Munch TA Effects of the jimpy mutation on mouse retinal structure and function. *J Comp Neurol*. 2015; 523: 2788–2806. 26011242 10.1002/cne.23818

[56] Mendoza-Santiesteban CE, Palma JA, Ortuno-Lizaran I, Cuenca N, Kaufmann H Pathologic confirmation of retinal ganglion cell loss in multiple system atrophy. *Neurology*. 2017; 88: 2233–2235. 28490649 10.1212/WNL.0000000000004020 PMC5467953

[57] Stefanova N, Reindl M, Neumann M, Kahle PJ, Poewe W, Wenning GK Microglial activation mediates neurodegeneration related to oligodendroglial alpha-synucleinopathy: implications for multiple system atrophy. *Mov Disord*. 2007; 22: 2196–2203. 17853477 10.1002/mds.21671

[58] Refolo V, Bez F, Polissidis A, et al. Progressive striatonigral degeneration in a transgenic mouse model of multiple system atrophy: translational implications for interventional therapies. *Acta Neuropathol Commun*. 2018; 6: 2. 29298733 10.1186/s40478-017-0504-y PMC5753576

[59] Gerhard A, Banati RB, Goerres GB, et al. [11C](R)-PK11195 PET imaging of microglial activation in multiple system atrophy. *Neurology*. 2003; 61: 686–689. 12963764 10.1212/01.wnl.0000078192.95645.e6

[60] Ramirez AI, de Hoz R, Salobrar-Garcia E, et al. The role of microglia in retinal neurodegeneration: Alzheimer's disease, Parkinson, and glaucoma. *Front Aging Neurosci*. 2017; 9: 214. 28729832 10.3389/fnagi.2017.00214 PMC5498525

[61] Ekstrom P, Sanyal S, Narfstrom K, Chader GJ, van Veen T Accumulation of glial fibrillary acidic protein in Müller radial glia during retinal degeneration. *Invest Ophthalmol Vis Sci*. 1988; 29: 1363–1371. 3417421

[62] Roy S Synuclein and dopamine: the Bonnie and Clyde of Parkinson's disease. *Nat Neurosci*. 2017; 20: 1514–1515. 29073642 10.1038/nn.4660

[63] Witkovsky P Dopamine and retinal function. *Doc Ophthalmol*. 2004; 108: 17–40. 15104164 10.1023/b:doop.0000019487.88486.0a

[64] Mendoza-Santiesteban CE, Palma JA, Martinez J, Norcliffe-Kaufmann L, Hedges TR 3rd, Kaufmann H Progressive retinal structure abnormalities in multiple system atrophy. *Mov Disord*. 2015; 30: 1944–1953. 26359930 10.1002/mds.26360 PMC4568758

[65] Polo V, Satue M, Rodrigo MJ, et al. Visual dysfunction and its correlation with retinal changes in patients with Parkinson's disease: an observational cross-sectional study. *BMJ Open*. 2016; 6: e009658. 10.1136/bmjopen-2015-009658 PMC4861131 27154474

[66] Ortuno-Lizaran I, Beach TG, Serrano GE, Walker DG, Adler CH, Cuenca N Phosphorylated alpha-synuclein in the retina is a biomarker of Parkinson's disease pathology severity. *Mov Disord*. 2018; 33: 1315–1324. 29737566 10.1002/mds.27392 PMC6146055

[67] Bodis-Wollner I, Miri S, Glazman S Venturing into the no-man's land of the retina in Parkinson's disease. *Mov Disord*. 2014; 29: 15–22. 24339212 10.1002/mds.25741

[68] Takeda K, Dezawa M, Kitada M The expression of PLP/DM-20 mRNA is restricted to the oligodendrocyte-lineage cells in the adult rat spinal cord. *Histochem Cell Biol*. 2016; 145: 147–161. 26563642 10.1007/s00418-015-1384-5

[69] Zhang C, Guo Y, Slater BJ, Miller NR, Bernstein SL Axonal degeneration, regeneration and ganglion cell death in a rodent model of anterior ischemic optic neuropathy (rAION). *Exp Eye Res*. 2010; 91: 286–292. 20621651 10.1016/j.exer.2010.05.021 PMC2907443

[70] Munemasa Y, Kitaoka Y Molecular mechanisms of retinal ganglion cell degeneration in glaucoma and future prospects for cell body and axonal protection. *Front Cell Neurosci*. 2012; 6: 60. 23316132 10.3389/fncel.2012.00060 PMC3540394

[71] Smith CA, Vianna JR, Chauhan BC Assessing retinal ganglion cell damage. *Eye (Lond)*. 2017; 31: 209–217. 28085141 10.1038/eye.2016.295 PMC5306472

[72] Nickells RW The cell and molecular biology of glaucoma: mechanisms of retinal ganglion cell death. *Invest Ophthalmol Vis Sci*. 2012; 53: 2476–2481. 22562845 10.1167/iovs.12-9483h PMC3990459

[73] Oueslati A Implication of alpha-synuclein phosphorylation at S129 in synucleinopathies: what have we learned in the last decade? *J Parkinsons Dis*. 2016; 6: 39–51. 27003784 10.3233/JPD-160779 PMC4927808

[74] Rodriguez L, Mdzomba JB, Joly S, Boudreau-Laprise M, Planel E, Pernet V Human tau expression does not induce mouse retina neurodegeneration, suggesting differential toxicity of tau in brain vs. retinal neurons. *Front Mol Neurosci*. 2018; 11: 293. 30197586 10.3389/fnmol.2018.00293 PMC6117378

[75] Wang X, Zhao L, Zhang J, et al. Requirement for microglia for the maintenance of synaptic function and integrity in the mature retina. *J Neurosci*. 2016; 36: 2827–2842. 26937019 10.1523/JNEUROSCI.3575-15.2016 PMC4879218

[76] Lopes da Fonseca T, Villar-Pique A, Outeiro TF The interplay between alpha-synuclein clearance and spreading. *Biomolecules*. 2015; 5: 435–471. 25874605 10.3390/biom5020435 PMC4496680

[77] Lee HJ, Khoshaghideh F, Patel S, Lee SJ Clearance of alpha-synuclein oligomeric intermediates via the lysosomal degradation pathway. *J Neurosci*. 2004; 24: 1888–1896. 14985429 10.1523/JNEUROSCI.3809-03.2004 PMC6730405

[78] Dice JF Peptide sequences that target cytosolic proteins for lysosomal proteolysis. *Trends Biochem Sci*. 1990; 15: 305–309. 2204156 10.1016/0968-0004(90)90019-8

[79] Cuervo AM, Stefanis L, Fredenburg R, Lansbury PT, Sulzer D Impaired degradation of mutant alpha-synuclein by chaperone-mediated autophagy. *Science*. 2004; 305: 1292–1295. 15333840 10.1126/science.1101738

[80] Xilouri M, Brekk OR, Landeck N, et al. Boosting chaperone-mediated autophagy in vivo mitigates alpha-synuclein-induced neurodegeneration. *Brain*. 2013; 136: 2130–2146. 23757764 10.1093/brain/awt131

[81] Armstrong RA Visual signs and symptoms of multiple system atrophy. *Clin Exp Optom*. 2014; 97: 483–491. 25256122 10.1111/cxo.12206

[82] Sartucci F, Porciatti V Visual-evoked potentials to onset of chromatic red-green and blue-yellow gratings in Parkinson's disease never treated with l-dopa. *J Clin Neurophysiol*. 2006; 23: 431–435. 17016154 10.1097/01.wnp.0000216127.53517.4d PMC3703931

[83] Langheinrich T, Tebartz van Elst L, Lagreze WA, Bach M, Lucking CH, Greenlee MW Visual contrast response functions in Parkinson's disease: evidence from electroretinograms, visually evoked potentials and psychophysics. *Clin Neurophysiol*. 2000; 111: 66–74. 10656512 10.1016/s1388-2457(99)00223-0

[84] Mammadova N, Summers CM, Kokemuller RD, et al. Accelerated accumulation of retinal alpha-synuclein (pSer129) and tau, neuroinflammation, and autophagic dysregulation in a seeded mouse model of Parkinson's disease. *Neurobiol Dis*. 2019; 121: 1–16. 30218757 10.1016/j.nbd.2018.09.013

[85] Krismer F, Wenning GK, Li Y, Poewe W, Stefanova N Intact olfaction in a mouse model of multiple system atrophy. *PLoS One*. 2013; 8: e64625. 23691255 10.1371/journal.pone.0064625 PMC3656866

