# Supplementary material for: Assessment of the Retina of Plp-α-Syn Mice as a Model for Studying Synuclein-Dependent Diseases
Source: Invest Ophthalmol Vis Sci. 2020 Jun 5;61(6):12. doi: 10.1167/iovs.61.6.12 (PMC7415298; doi:10.1167/iovs.61.6.12)
Supplement: Supplement 1 [file iovs-61-6-12_s001.pdf]

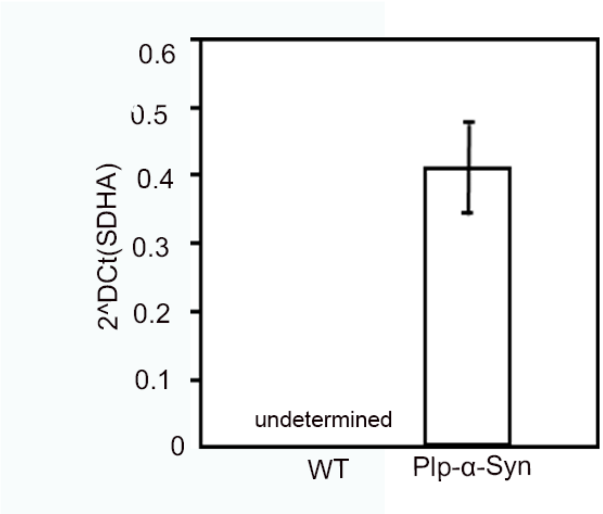

Supplementary figure 1: Human α-Syn in wild type and Plp-α-Syn retinas. Expression levels of human α-Syn mRNA. Data are presented as means ± SEM (N = 3 for wild type (WT) and N = 4 for Plp-α-Syn retinas). Ct values were normalized to the housekeeping gene SDHA: mean ± SEM for Plp-α-Syn: 0.42 ±0.06 for N = 5; CT values for wild type were undetermined. The age of animals was 8-10 weeks.
