# Supplementary material for: Assessment of the Retina of Plp-α-Syn Mice as a Model for Studying Synuclein-Dependent Diseases
Source: Invest Ophthalmol Vis Sci. 2020 Jun 5;61(6):12. doi: 10.1167/iovs.61.6.12 (PMC7415298; doi:10.1167/iovs.61.6.12)
Supplement: Supplement 2 [file iovs-61-6-12_s002.pdf]

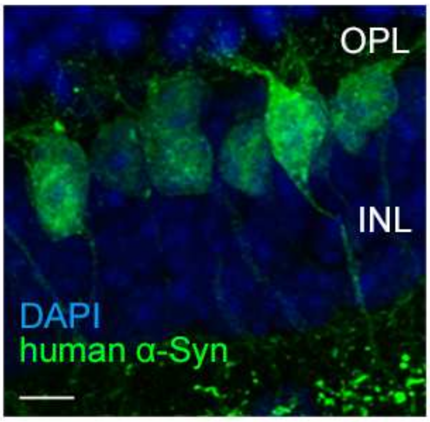

Supplementary figure 2: Human α-Syn in rod bipolar cells. Human α-Syn (green) in rod bipolar cells of Plp-α-Syn mice in high resolution. Their cell bodies are typically closest to the outer plexiform layer (OPL) Scale bar: 10 µm. The age of animals was 8 - 10 weeks. Other abbreviation: inner nuclear layer (INL).
