# Supplementary material for: Assessment of the Retina of Plp-α-Syn Mice as a Model for Studying Synuclein-Dependent Diseases
Source: Invest Ophthalmol Vis Sci. 2020 Jun 5;61(6):12. doi: 10.1167/iovs.61.6.12 (PMC7415298; doi:10.1167/iovs.61.6.12)
Supplement: Supplement 3 [file iovs-61-6-12_s003.pdf]

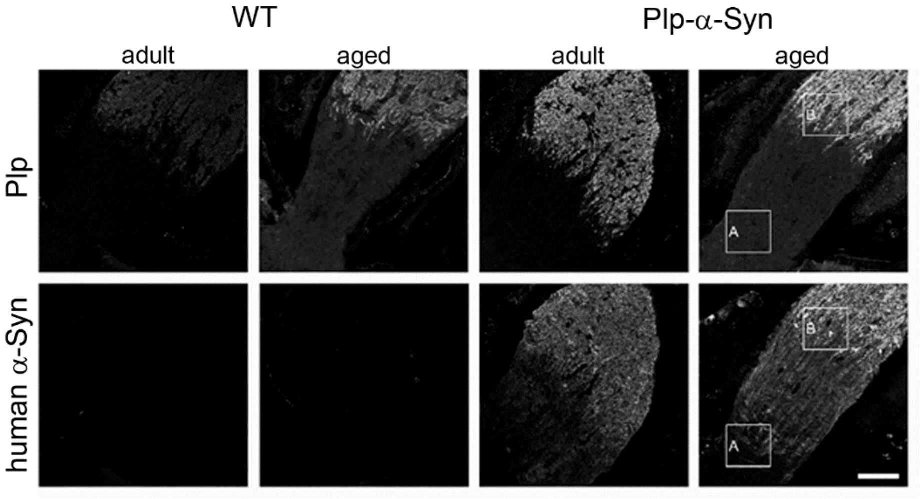

Supplementary figure 3: Maximum projections of co-labelling of Plp and human α-Syn in the optic nerve. Adult and aged wild type (WT) and Plp-α-Syn animals were compared at the age of 8 – 10 weeks (adult) and 12 months (aged). Colocalisation of the two signals tested in individual slices of the image stack in two regions of interest: A corresponds to the area where the optic nerve enters the retina and B covers a more distal region on the optical nerve. For quantification, see Supplementary table. Scale bar: 75 µm.
