# Supplementary material for: Assessment of the Retina of Plp-α-Syn Mice as a Model for Studying Synuclein-Dependent Diseases
Source: Invest Ophthalmol Vis Sci. 2020 Jun 5;61(6):12. doi: 10.1167/iovs.61.6.12 (PMC7415298; doi:10.1167/iovs.61.6.12)
Supplement: Supplement 4 [file iovs-61-6-12_s004.pdf]

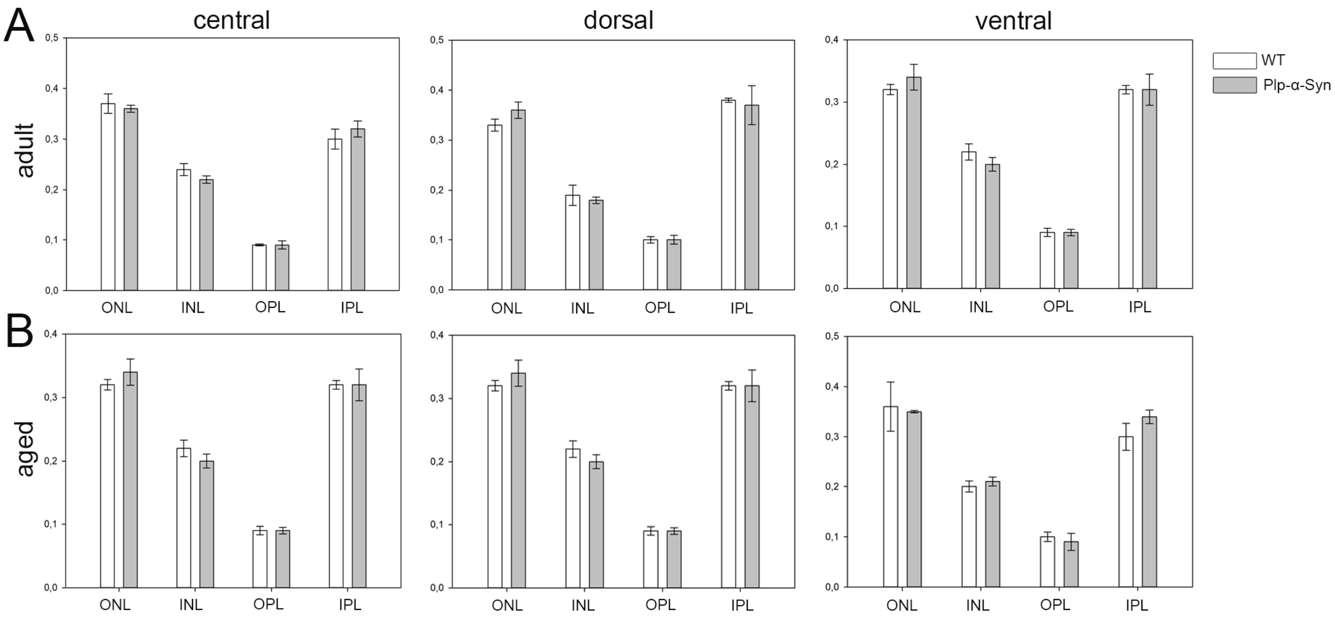

Supplementary figure 4: Relative layer thickness in central, dorsal and ventral areas of the retina. **(A)** The figure depicts data from adult (8-10 weeks) mice, **(B)** from aged (12 months) mice for both groups: wild type (WT) and Plp-α-Syn. Data for each layer normalized to the individual total retinal thickness. Data are shown as means ± SEM. N = 3, for each group (except for aged animals, central N = 4). Statistics: Student's t-test with Holm-Bonferroni post-test. Abbreviations: outer nuclear layer (ONL), outer plexiform layer (OPL), inner nuclear layer (INL), inner plexiform layer (IPL).
