# Supplementary material for: Assessment of the Retina of Plp-α-Syn Mice as a Model for Studying Synuclein-Dependent Diseases
Source: Invest Ophthalmol Vis Sci. 2020 Jun 5;61(6):12. doi: 10.1167/iovs.61.6.12 (PMC7415298; doi:10.1167/iovs.61.6.12)
Supplement: Supplement 5 [file iovs-61-6-12_s005.pdf]

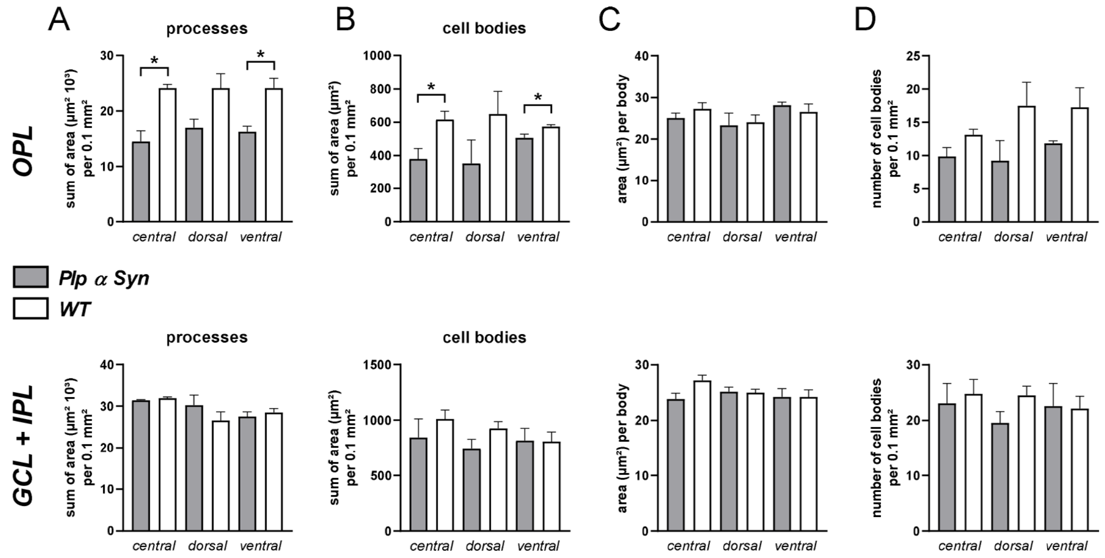

Supplementary figure 5. Quantification of Iba1⁺ microglia cells. Images of Iba1⁺ microglial cells in central, dorsal and ventral retinas of wild type (WT) and Plp-α-Syn mice were segmented. Quantification of Plp-α-Syn or WT microglia in OPL (top row) or GCL and IPL (bottom row). The sum of all segmented areas of processes **(A)** or cell bodies **(B)** per 0.1 mm² were measured. Furthermore, the area per cell body **(C)** and the number of cell bodies per 0.1 mm² **(D)** was quantified. Significant differences between Plp-α-Syn and WT within central, dorsal or ventral locations were calculated with the Student's t-test or, when normality criteria were not met, a test on ranks was performed ($p < 0.05$). Significant overall effects in the OPL between the two mouse models were also detected when performing a 2-Way ANOVA (factor 1: model, factor 2: location) in panels A, B, and D. Data are shown as means ± SEM, N = 3 - 5. * $p<0.05$.
