# Supplementary material for: Assessment of the Retina of Plp-α-Syn Mice as a Model for Studying Synuclein-Dependent Diseases
Source: Invest Ophthalmol Vis Sci. 2020 Jun 5;61(6):12. doi: 10.1167/iovs.61.6.12 (PMC7415298; doi:10.1167/iovs.61.6.12)
Supplement: Supplement 6 [file iovs-61-6-12_s006.pdf]

Supplementary Table: Colocalisation indices of Plp and human α-Syn in the optic nerve. Adult (8 – 10 weeks) and aged (12 months) WT and Plp-α-Syn animals. ROI A corresponds to the area where the optic nerve enters the retina and ROI B covers a more distal region on the optical nerve (Supplementary figure 2). Values with redish filling indicate absence of colocalisation, whereas greenish labelling supports colocalisation. [1] Pearson's correlation (-1 = complete anticolocalisation, 0 = noncolocalisation, 1 = complete colocalisation); [2] Intensity Correlation Quotient (ICQ) (-0.5 = complete anticolocalisation, 0 = noncolocalisation, 0.5 = complete colocalisation); [3] Manders' Colocalisation Coefficient (0 = complete anticolocalisation, 1 = complete colocalisation); [4] Spearman's Rank Correlation Coefficient (-1 = complete anticolocalisation, 0 = noncolocalisation, 1 = complete colocalisation).

	WT adult		WT aged		Plp α-Syn adult		Plp α-Syn aged	
ROI	A	B	A	B	A	B	A	B
Pearson's R value [1]	0.19	0.13	0.36	0.25	0.28	0.70	0.39	0.74
Li's ICQ value [2]	0.15	0.13	0.21	0.16	0.16	0.33	0.22	0.35
Manders' tM1 (above auto- threshold of anti-Plp) [3]	0.36	0.15	0.80	0. 38	0.57	0.88	0.89	0.92
Manders' tM2 (above auto- threshold of anti-human α-Syn) [3]	0.29	0.29	0.39	0.40	0.43	0.87	0.90	0.92
Spearman's rank correlation value [4]	0.52	0.27	0.71	0.50	0.44	0.73	0.66	0.79
